# Accumulation of lipids after acute direct and indirect traumatic injuries in male and female mice

**DOI:** 10.1186/s12891-025-09207-5

**Published:** 2025-10-08

**Authors:** Angela S. Bruzina, Braydon A. Crum, Christiana J. Raymond-Pope, Jarrod A. Call, Sarah M. Greising

**Affiliations:** 1 School of Kinesiology, University of Minnesota, 1900 University Ave SE, Minneapolis, MN 55455 USA; 2 Department of Physiology and Pharmacology, University of Georgia, Athens, GA 30602 USA; 3 Regenerative Bioscience Center, University of Georgia, Athens, GA 30602 USA

**Keywords:** Adipokine, Denervation, In vivo muscle function, Volumetric muscle loss

## Abstract

**Background:**

Adipose infiltration and lipid droplet accumulation are implicated in metabolic diseases and are known to limit skeletal muscle repair and regeneration. However, their role in skeletal muscle injury, particularly volumetric muscle loss (VML), remains unclear. We aimed to characterize early lipid and adipokine responses following direct (i.e., VML) and indirect (i.e., denervation) traumatic injuries.

**Methods:**

Adult male and female C57Bl/6J mice (*n* = 36) were randomized to VML injury to the posterior hindlimb, tibial nerve denervation, or remained injury Naïve. Three days post, in vivo muscle function was assessed. Serum and gastrocnemius muscle tissue were assessed for histological and biochemical analyses.

**Results:**

A decrease in total myofiber number occurred post-VML with a reduction in force and increased fatigue. Lipid droplet-associated proteins displayed injury- and myofiber type-specific changes, with VML exhibiting accumulation of perilipin 5 localized to the injury site. Lipid droplets in the muscle remaining were significantly greater post-VML compared to denervation. The pro-inflammatory adipokine IL-6 and growth factor IGF-1 were markedly increased in the muscle remaining post-VML, while circulating metabolic regulators, adiponectin and leptin, were suppressed.

**Conclusions:**

These findings underscore lipid droplet dynamics and adipokine signaling disruptions following indirect and direct traumatic injuries in a preclinical model. Future work should be done to explore aspects of lipid droplet regulation temporally following skeletal muscle injuries, as early accumulation may suggest an additional etiology in the pathological sequelae of injury.

**Supplementary Information:**

The online version contains supplementary material available at 10.1186/s12891-025-09207-5.

## Background

Lipid droplets, commonly recognized as the storage units of adipocytes, play a dynamic physiological role in skeletal muscle by mediating energy homeostasis. Physiologically, lipid droplets release free fatty acids to serve as substrates for mitochondrial β-oxidation in response to an increase in energy demands. Although lipid droplets play a critical role in maintaining energy balance, they can become dysregulated (e.g., hypertrophy) under pathological conditions, resulting in accumulation within the cellular space of myofibers. Recently, infiltration of adipocytes between individual myofibers has been characterized in cardiometabolic disorders, such as obesity and type 2 diabetes [1–3]. Adipocytes release signaling proteins, known as adipokines, which can regulate metabolism, inflammation, and lipid droplet dynamics [4–6]. There is an emerging link between lipid accumulation and functionality of skeletal muscle; specifically, increases in lipid accumulation are associated with impairments in muscle contractile force and increased fatigue [7, 8]. In response to physiological stimuli, such as skeletal muscle injury or stress, lipid droplets hypertrophy to store excess lipid intermediates resulting from cellular dysfunction [9, 10]. It has been suggested that the regenerative capacity of skeletal muscle could, in part, be impaired by pathological adipocyte and lipid droplet accumulation [11, 12].

Adipokines such as adiponectin and leptin modulate the post-injury response to traumatic skeletal muscle injury. Following injury, repair and regeneration of skeletal muscle is mediated by integrated cellular signaling, including interactions between injured myofibers, resident cells, and adipose tissue [13]. The rapid release of pro-inflammatory cytokines, for example interleukin-6 (IL-6) and monocyte chemoattractant protein-1 (MCP-1), post-injury facilitates recruitment of immune cells and promotes macrophage-mediated clearing of damaged tissue. Prolonged and exacerbated inflammatory signaling contributes to lipid accumulation within skeletal muscle from dysfunctional cellular metabolism and ineffective tissue remodeling [14, 15]. This inflammatory milieu also promotes increased droplet-associated proteins, such as perilipin 2. Perilipin 2 contributes to lipid accumulation within the cellular space of myofibers, which triggers the localized release of adipokines within skeletal muscle. The inflammatory response of the adipokines is known to inhibit the canonical muscle protein synthesis pathway (IGF-1/Akt/mTOR) leading to the promotion of lipid mobilization. However, to date, few studies have explored how sever acute injury affects these adipokines, particularly in relation to lipid accumulation or mobilization.

Volumetric muscle loss (VML) is a traumatic skeletal muscle injury that results in tissue losses that occur either directly from the direct mechanism of injury itself, or secondarily as a result of surgical procedures performed during limb salvage procedures. Volumetric muscle loss injury results in the frank loss of contractile tissue and is characterized by limited regenerative potential and chronic functional deficits [16, 17]. Temporal changes in whole-body metabolism occur following VML with alterations in lipid oxidation and diurnal metabolic flexibility [18–20]. These changes are reported as potential prognostic indicators of metabolic dysfunction and the development of metabolic disease [21, 22]. Limited skeletal muscle regeneration following VML is partly attributed to an exacerbated inflammatory response [23–25] and mitochondrial metabolism dysfunction [26–29]. This dysfunction can influence cellular lipid dynamics, as injury may be impairing the necessary interaction between mitochondria and lipid droplets [30, 31] Though VML exhibits cellular characteristics associated with pathological lipid droplets, there is currently a lack of work exploring this concept. Injuries that indirectly impact the skeletal muscle, such as denervation, can also result in decreased functional capacity due to the loss of myofibers and atrophy of existing myofibers [32]. Following denervation, the limited regenerative response is disrupted by sustained pro-inflammatory gene expression and reactive oxygen species production [33]. Lipid accumulation has been demonstrated as early as 7-days following denervation [34], emphasizing the need for earlier evaluation of lipid-associated signaling.

Understanding lipid accumulation and signaling in the context of skeletal muscle injuries, particularly those associated with the development of metabolic disease, cellular disruption and functional impairments, is warranted. This work was designed to characterize regulatory pathways and signaling protein (e.g., adipokine) response both locally and systemically following injury. Comparison was made between a direct and indirect muscle injury, VML to the posterior compartment or tibial nerve denervation, respectively, at an early time point (i.e., 3 days post). Understanding early signaling may provide valuable insights into initial cellular responses that shape downstream tissue remodeling, lipid accumulation, and systemic metabolic consequences. The early post-injury time point captures initial disruption of critical skeletal muscle components, including the extracellular matrix and remodeling-associated cells (e.g., fibroadipogenic progenitors, satellite cells), thereby enabling evaluation of regenerative or pathological remodeling [35, 36]. Identifying unique or shared regulatory mechanisms that may contribute to chronic changes to the function and structure of skeletal muscle following injury may inform acute therapeutic interventions.

## Methods

### Ethical approval

All protocols were approved by the Institutional Animal Care and Use Committee at the University of Minnesota (2107–39253 A) in compliance with the Animal Welfare Act, the Implementing Animal Welfare Regulations and in accordance with the principles of the Guide for the Care and Use of Laboratory Animals.

### Study design

Adult male and female C57B1/6J (*n* = 36) were purchased from Jackson Laboratories (Stock #000664; Bar Harbor, ME). Mice were housed on a 12-hr light-dark cycle with *ad libitum* access to chow and water. Mice were allowed at least one-week of acclimation before any experimentation. At ~ 12.5 weeks of age, mice were randomly assigned to VML to the posterior compartment or tibial nerve denervation experimental groups, or a Naïve non-injured, age- and sex-matched control group (*n* = 12; 6 male, 6 female). Mice were provided *ad libitum* access to chow and water leading up to any terminal experiments or sample collection. Terminally, 3 days following injury, in vivo muscle function was assessed. Gastrocnemius muscles and liver tissue were harvested, frozen, and stored at −80℃ for biochemical or histological analyses. Serum was collected, prepared, and stored at −20℃ for immunoassay analyses. Mice were euthanized with intraperitoneal pentobarbital (> 100 mg/kg).

### Surgical creation of volumetric muscle loss (VML) and denervation injury

For both surgical procedures, buprenorphine-SR (1.0 mg/kg s.q.) was administered 2 h prior to surgery for pain management. Surgeries were performed under aseptic conditions, while mice were anesthetized by isoflurane inhalation (1–3%). Depth of anesthesia was monitored and adjusted accordingly. Any bleeding was controlled with light pressure and all skin incisions were closed with non-absorbable suture. Animals were monitored through recovery.

A full thickness VML injury was surgically created in the posterior compartment muscle group (gastrocnemius, soleus, plantaris muscles) as previously described [29, 37, 38]. Briefly, the gastrocnemius muscle was exposed via a posterior-lateral incision (~ 2 cm) through the skin. To isolate the posterior muscle compartment, blunt dissection was used to isolate the muscle compartment off the dorsal aspect of the tibia. A small metal plate was inserted between the tibia and the deep aspect of the soleus. A 4 mm punch biopsy removed the middle third of the muscle compartment (males 18.8 ± 0.8 mg; females 14.7 ± 2.0 mg). Sutures were placed to identify the edges of the excised area on the gastrocnemius muscle at the proximal and distal point.

Denervation was conducted by severing the tibial nerve, denervating ankle plantarflexor muscles as previously described [39, 40]. Briefly, the bicep femoris was exposed via a ~ 15 mm incision through the skin, slightly posterior to the femur. An incision was made through the biceps femoris muscle, and the flaps were retracted to expose the sciatic, common peroneal, and tibial nerves. The tibial nerve was specifically located and isolated dissected away from the musculature and fat, then was severed just distally to the branch from the sciatic nerve. An ~ 3 mm gap of the nerve was removed. The bicep femoris muscle flaps were sutured together, and skin was closed.

### In vivo muscle function

Muscle function of the posterior compartment was determined terminally 3 days following injury as previously described [19, 20, 37, 41]. Briefly, mice were anesthetized with isoflurane (1–2%) and positioned on the right side. The left knee and hip were stabilized at 90⁰ with a knee clamp, and the left foot was attached to the footplate of a dual-mode muscle lever system (300 C-LR; Aurora Scientific, Aurora, Ontario, Canada). Maximal isometric torque was evaluated after severing the common peroneal nerve to avoid stimulation of the anterior compartment. Sciatic nerve stimulation was performed using Platinum-Iridium percutaneous needle electrodes. After achieving optimal nerve stimulation, twitch torque was obtained at 5 Hz and maximal isometric torque was obtained at 150–200 Hz. Torque is expressed as mN·m per kg body weight. Following a 3-minute rest, a 3-minute fatiguing protocol was implemented consisting of a 60-Hz stimulation every 2 s for a total of 90 contractions. A fatigue index was calculated as the percentage of torque decline from the first to the final contraction. Due to the nature of the denervation injury to the tibial nerve hindering the ability of the posterior compartment muscles to produce force, only a subset of denervation-injured mice underwent in vivo muscle function testing procedures. With a confirmed ~ 99% reduction in maximal torque production, denervation-injured mice were not included in contractile analyses.

### Biochemical analyses

Terminally, the gastrocnemius muscle was harvested and cut into thirds including the proximal, middle, and distal regions of the muscle, with the middle portion containing the VML defect region. The proximal and middle portions were used for biochemical analyses. A portion of harvested liver tissue was also saved for biochemical analyses. Total protein content was analyzed using the Protein A280 setting on a NanoDrop One spectrophotometer (Thermo Scientific) in triplicate and averaged. The middle portion of muscle, which contained the defect, and the liver were weighed and homogenized in a phosphate buffer at a ratio of 1:5 and 1:10 (mg/µl), respectively. The proximal portion of muscle was weighed and homogenized in RIPA buffer at a ratio of 1:10 (mg/µl). All homogenates were processed with protease inhibitors at 1:100 (mg/µl). Blood collected terminally was centrifuged at 2,000 rpm for 15 min at 4℃. Clear separation between serum and clotted blood cells were confirmed prior to collection of serum.

For immunoblot analyses, 30–50 µg of protein were separated by 4–15% Criterion TGX Stain-Free Gel, transferred onto a low fluorescence PVDF membrane, and immunoblotted. The following primary antibodies were used to detect protein quantities within the middle or proximal portions of the muscle: Adiponectin (Cell Signaling Technology Cat# 2789; RRID: AB_2221630; 10 µg/ml), PDGFRα (R&D Systems Cat# AF1062; RRID: AB_2236897; 3 µl/ml), Follistatin (R&D Systems Cat# AF669; RRID: AB_2247223; 0.3 µl/ml), Perilipin 5 (Proteintech Cat#26951-1-AP; RRID: AB_2880699; 1000 µl/ml), and Perilipin 2 (Proteintech Cat#15294-1-AP; RRID: AB_2878122; 1000 µl/ml). Primary antibodies were detected using a host- and isotype-specific horseradish peroxidase conjugated secondary antibody as follows: Rabbit anti-goat IgG HRP (Abcam Cat# 6741; RRID: AB_955424, 1:10,000), anti-rabbit IgG HRP (Cell Signaling Technology Cat# 7074; RRID: AB_2099233, 1:1000). Immunoblots were blocked and incubated with primary and secondary antibodies with 5% milk or bovine serum albumin in 0.1% Tween TBS. Immunoblots were imaged with stain-free chemiluminescence using a ChemiDoc MP System (Bio-Rad Laboratories, Hercules, CA, USA). The band of interest was identified per the manufacturer-suggested molecular weight. Total protein was quantified in each lane and the intensity of each band was normalized to total protein in each respective lane. The gastrocnemius of injury-Naïve control mice was used for comparison and all experimental samples were normalized to the control during analysis. All uncropped gels and blots are reported in Supplemental Fig. 1.

A multiplex assay was used to assess various adipokine and inflammatory markers in the serum and the middle portion of the gastrocnemius muscle, specifically, IL-6, TNF-a, MCP-1, Insulin, Leptin (Milliplex MADKMAG-71 K). Plates were prepared according to manufacturer protocol and read using a Bio-Plex-200 system (Bio-Rad; Hercules, CA, USA). Prepared standards were excluded from the standard curve if they were outside an acceptable recovery range (observed concentration/expected concentration) of 70–120%. Individual analytes were excluded from the analysis if less than 80% of the total samples were below the minimum detectable concentration; MCP-1 and TNF-a concentrations from serum and muscle did not meet this threshold and were omitted from the analysis. An ELISA immunoassay was used to evaluate insulin-like growth factor (IGF-1) across the middle portions of the gastrocnemius muscle, serum, and liver tissue (R & D Systems Cat# MG100). Muscle and liver tissue were diluted 1:5 (mg/µl) and 1:40 (mg/µl) respectively. Samples were analyzed according to manufacturer protocol and normalized to protein content during analysis. Sample concentrations were excluded from immunoassay analyses if the observed concentration was outside the range of valid standards.

### Histological analyses and imaging

In a subset of 4 mice per group, the whole gastrocnemius muscle was harvested and placed in OCT prior to freezing in isopentane cooled by liquid nitrogen. Gastrocnemius muscles were stored at −80℃ until histological processing. Systematic 10 μm serial cross-sections were obtained across five regions along the entire length of the gastrocnemius muscle, as previously described [42]. Briefly, the whole muscle was cut proximal to distal. The first ~ 100 μm was cut from the proximal end of the muscle (P1) where 10 cross-sections were collected. Ten cross-sections were then collected every 1.5 mm interval for a total of 5 distinct sections across the full length of the gastrocnemius muscle (P1, P2, Mid, D1, D2). This strategy aligns the middle region (Mid) sectioned with the mid-belly of the gastrocnemius and thus the center of the VML defect region. Systematically obtained muscle sections were stained with hematoxylin and eosin for histological analyses of total myofiber number and with Oil-Red-O for lipid percent area quantification. Brightfield images were acquired on the TissueScope LE slide scanner (Huron Digital Pathology, St. Jacobs, ON, Canada) using a 20X objective (0.75 NA, 0.5 μm/pixel resolution). Myofibers were manually counted using the multipoint tool in FIJI [43].

Within the middle muscle section only, the muscles were stained for BODIPY, an additional marker for lipid droplet accumulation. Briefly, sections were removed from − 80℃ storage and allowed to fully thaw and dry (~ 30 min). Sections were incubated in anti-laminin (Abcam Cat# ab11575; RRID: AB_298179; 7 µg/ml) in PBS for 1 h at 25 °C before being washed in PBS. Sections were then incubated in a host- and iso-type specific secondary antibodies (Thermo Fisher Scientific Cat# A21245; RRID: AB_2535813; 20 µg/ml) and DAPI (Thermo Fisher Scientific Cat# D21490; 10 µg/ml) in PBS for 1 h at 25 °C, then washed in PBS. Finally, sections were incubated in BODIPY (Thermo Fisher Scientific Cat# D3922; 0.5 mg/ml) in DMSO for 30 min at 25 °C, before a final wash in PBS. Sections were air dried, and cover slipped with Prolong Diamond Antifade Mountant (Thermo Fisher Scientific Cat# P36970). The sections stained with BODIPY were captured on a Nikon AXR confocal microscope using a resonant scanner and Plan Apo 20X objective (0.8 NA, DIC N2). The whole tissue was imaged by taking 20X frames (2048 × 2048 pixels; 0.43 μm/pixel) with 15% overlap. Each frame was then stitched together using Nikon NIS-Elements AR software and analysis was completed on the full stitched image. The lipid percent area from both Oil-Red-O brightfield and BODIPY fluorescent staining was collected using threshold and ROI manager tools in FIJI [43].

Within the middle muscle section only, muscles were stained for myosin heavy chain (MyHC) isoform expression MyHC_slow_ (DSHD Cat# BA-D5, RRID: AB_2235587; 5 µg/ml) and MyHC_2A_ (DSHB Cat# SC-71; RRID: AB_2147165; 5 µg/ml) in conjunction with perilipin 5 (Proteintech Cat#26951-1-AP; RRID: AB_2880699; 5 µg/ml) to analyze changes in fiber type, perilipin 5 expression and co-localization. Images (1192 × 1192 pixels; 0.46 μm/pixel) were acquired on a Nikon Eclipse Ti widefield fluorescence microscope using a 20X objective (Plan Fluor, 0.5 NA) from standardized lateral, medial, border, and defect regions for the muscle tissue, as previously described [19]. Across each region of interest, myofibers were classified as type I, IIa, IIb and/or IIx based on stating of MyHC_slow_, or MyHC_2A_, or by the absence of staining for type IIb and/or IIx, respectively. Fiber type count and average cross-sectional area were also analyzed using Nikon NIS-Elements AR software. Using FIJI, the percent area of positively stained tissue expressing Perilipin 5 in all three fiber types, combined fibers, and total image area was quantified. Only perilipin 5 that was co-localized within myofiber boundaries was included in fiber type specific quantifications, perilipin 5 not co-localized in boundaries was only included in in the total area. Investigators were blinded during all imaging and post-imaging analyses.

### Statistical analyses

All data were analyzed using GraphPad Prism (version 10.3.1, GraphPad Software, San Diego, CA, USA) or JMP software (version 17.0 SAS Institute, Cary, NC, USA). Two-way ANOVAs (injury x sex) with Tukey’s HSD *post hoc* evaluated differences in masses, maximal isometric torque, fatigue index, and contractile parameters (i.e., rates of contraction and relaxation and time to maximal contraction), protein content (e.g., adiponectin), circulating factors (e.g., IGF-1), and histologic outcomes (i.e., percent area of lipids, perilipin 5, cross-sectional area, and fiber type distribution). Three-way ANOVA was used to assess myofiber number across muscle region (injury x sex x whole muscle region) with Tukey’s HSD *post hoc* to evaluate interactions. Significance was set at *p* ≤ 0.05. All p-values and statistical tests conducted are reported in Supplemental Table 1.

## Results

### Animals

**Fig. 1:**
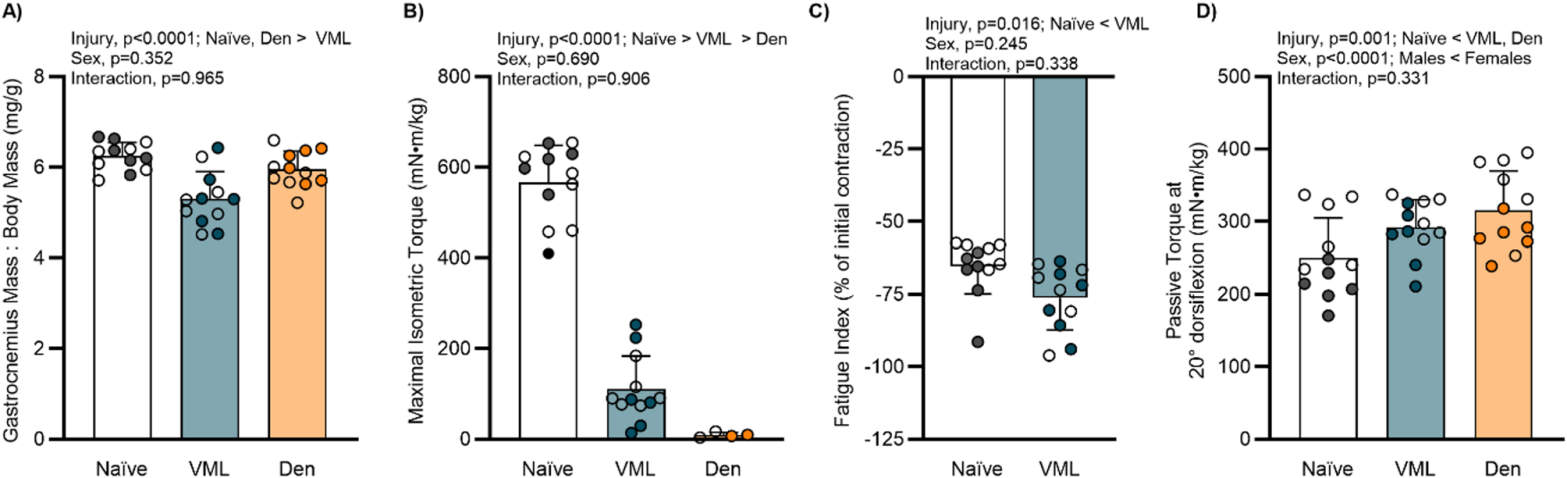
Functional assessment of remaining muscle following injury. **A** Terminally, VML-injured gastrocnemius muscles normalized to body mass were smaller than non-injured muscles (main effect of injury *p* < 0.0001). **B** Tibial nerve denervation eliminated the ability of the posterior compartments to produce force, and similarly VML injury significantly reduced maximal isometric torque (main effect of injury *p* < 0.0001; only a subset of denervated muscles were evaluated as confirmation of injury). **C** The percentage of torque lost after a muscle fatiguing protocol was greater in VML-injured muscles than in injury Naïve muscles (main effect of injury *p* = 0.016). **D** VML and denervation injuries significantly increased passive torque about the ankle joint at 20° dorsiflexion, which was more pronounced in females (main effect of injury *p* = 0.001; sex *p* < 0.0001). Open circle female, closed male; dots represent an individual animal. Data analyzed by two-way ANOVA; statistically significant Main Effects are noted; all p-values are shown within each graph and in Supplemental Table 1

Mice were randomized into VML or denervation injury groups or were age- and sex- matched injury Naïve controls. All mice that underwent surgical procedures recovered promptly without complication. As expected, female mice were smaller than males at ~ 12 weeks of age, both initially and terminally (Table 1). Terminally, 3 days after injury, mice that underwent both VML and denervation had less body mass than injury Naïve controls (Table 1). However, when accounting for body mass, only the VML injury resulted in smaller gastrocnemius muscles (Table 1; Fig. 1A).

**Table 1:** Body and muscle masses

	Male			Female			Two-way ANOVA *p*-value		
	Injury Naïve	VML	Denervation	Injury Naïve	VML	Denervation	Main Effect Injury	Main Effect Sex	Interaction
	*n* = 6	*n* = 6	*n* = 6	*n* = 6	*n* = 6	*n* = 6			
Initial Body Mass (g)	28.1 ± 1.5	27.4 ± 1.2	27.3 ± 1.8	20.8 ± 1.6	19.5 ± 1.6	19.3 ± 1.7	0.168	< 0.0001	0.854
Terminal Body Mass (g)	28.1 ± 1.5	26.2 ± 1.2*	25.9 ± 2.3*	20.7 ± 1.6	18.4 ± 0.9*	18.4 ± 1.4*	0.001	< 0.0001	0.902
Tibialis Anterior Mass: Body Mass (mg/g)	1.98 ± 0.15	1.99 ± 0.11	1.85 ± 0.17	2.02 ± 0.15	1.97 ± 0.09	1.87 ± 0.22	0.078	0.763	0.871
Gastrocnemius Mass: Body Mass (mg/g)	6.31 ± 0.32	5.35 ± 0.67	6.06 ± 0.34	6.17 ± 0.32	5.25 ± 0.58	5.86 ± 0.45	< 0.0001	0.352	0.841
Gastrocnemius Protein Content (mg/ml)	15.03 ± 0.79†	14.45 ± 0.63†	16.12 ± 1.04†	15.04 ± 0.59†	15.04 ± 1.05†	18.22 ± 0.39	---	---	0.043

Data mean ± SD

Significant main effect different than *Injury Naïve

Significant interaction different than †Female Denervation

### Impact of acute injury on muscle function

Initial loss of muscle function following injury is not able to predict long term recovery of function. Various muscle injuries (e.g., eccentric contraction- or toxin-induced) are known to fully recover muscle function long-term [44, 45], while muscle function following VML demonstrates chronic long-term deficits [41, 46, 47]. As expected, the indirect injury tibial nerve denervation obliterated the capacity of the posterior hindlimb compartment to produce force 3 days following denervation, confirming complete denervation of the tibial nerve was achieved (main effect of injury *p* < 0.001; Fig. 1B). Following VML injury, there was a dramatic decline in maximal isometric torque at 3 days (Fig. 1B), corresponding with substantially lower average rates of contraction and relaxation that were more pronounced in females (main effect of injury *p* < 0.0001; sex *p* ≤ 0.005; Table 2). In response to a fatiguing protocol, the muscle’s ability to produce force was significantly reduced following VML, independent of sex (main effect injury *p* = 0.016; Fig. 1C). In contrast, VML-injured and denervated muscles had increased passive torque as early as 3 days following injury; this response was heightened in females (main effect of injury *p* = 0.001; sex *p* < 0.0001; Fig. 1D). Observations confirm the immediate detriment of VML and denervation injuries on the ability of the muscle to produce force and be compliant to passive stretch; additionally, VML injury contributes to an inability to resist contraction-induced fatigue.

**Table 2:** In vivo muscle function and contractile characteristics

	Male			Female			Two-way ANOVA *p*-value		
	Injury Naïve	VML	Denervation	Injury Naïve	VML	Denervation	Main Effect Injury	Main Effect Sex	Interaction
	*n* = 6	*n* = 6	*n* = 6	*n* = 6	*n* = 6	*n* = 6			
60 Hz/Peak (%)	0.85 ± 0.06	0.83 ± 0.12	---	0.85 ± 0.05	0.88 ± 0.09	---	0.830	0.443	0.472
Time to Peak Force (s)	0.084 ± 0.023	0.098 ± 0.065	---	0.087 ± 0.022	0.087 ± 0.046	---	0.317	0.655	0.870
½ Relaxation Time (s)	0.047 ± 0.007	0.067 ± 0.039	---	0.047 ± 0.006	0.054 ± 0.008	---	0.144	0.513	0.570
+dP/dt (mN·m/s)	282.4 ± 77.5	57.8 ± 35.6	---	189.0 ± 27.0	39.6 ± 16.8	---	< 0.0001	0.003	0.257
-dP/dt (mN·m/s)	−379.3 ± 76.7	−53.0 ± 40.1	---	−277.2 ± 38.5	−37.3 ± 12.2	---	< 0.0001	0.005	0.175

Data mean ± SD

### Acute histologic changes to the gastrocnemius muscle

**Fig. 2:**
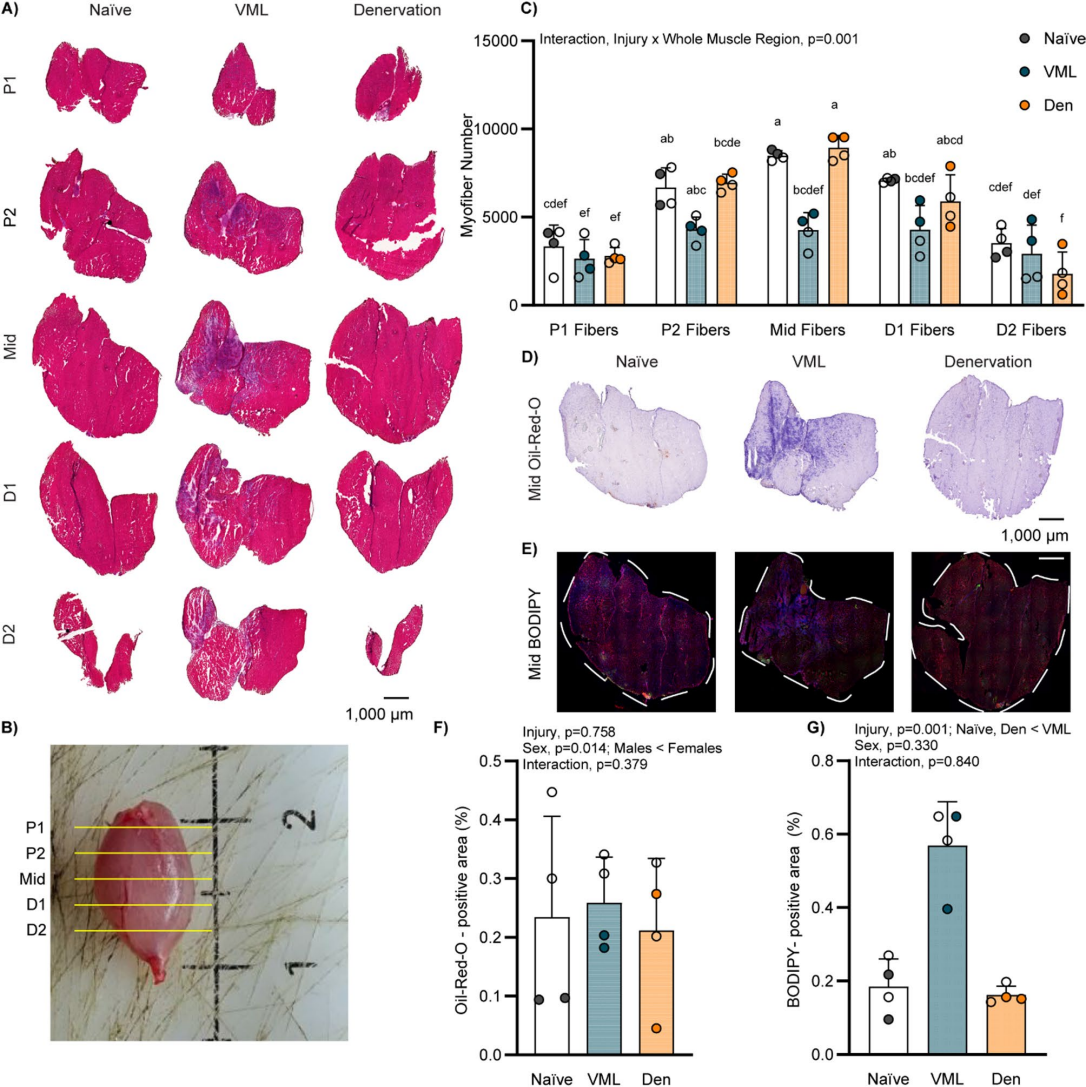
Systematic histological analysis of whole gastrocnemius muscle. **A** Representative H&E-stained images of systematically sectioned whole gastrocnemius muscle for all three groups shown proximal (P1 & P2) to distal (D1 & D2); noted these are the “whole muscle regions”. **B** Schematic of whole muscle sectioning technique. **C** Myofiber number across systematic sections of whole gastrocnemius (interaction injury x whole muscle region *p* = 0.001). The mid-belly section (mid) of each muscle was stained for **D** oil-red-O and **E** BODIPY (green), laminin (red), and DAPI (blue); scale bar is equal to 1,000 μm; **A**,** D**, and **E** and the mid-section displayed represents sequential sections in the same muscle. The lipid positive area was analyzed independently for lipid deposition as a percentage of the whole muscle region; with quantification for both analyses of **F** oil-red-O (main effect of sex *p* = 0.014), and **G** BODIPY (main effect of injury *p* = 0.001). Open circle female, closed male; dots represent an individual animal. Data for myofiber number in **C** analyzed by three-way ANOVA for injury x sex x whole muscle region. Significant differences within a region only are designated if they do not share the same letter. Data for lipid area in **F**&**G** analyzed by two-way ANOVA, statistically significant Main Effects are noted; All p-values are shown within each graph and in Supplemental Table 1

In a subset of muscles, systematic histological evaluation of the whole gastrocnemius was performed to characterize changes in the remaining muscle following injury (Fig. 2A&B). As frank loss of skeletal muscle is the primary mechanism of VML injury (18.5 mg and 14.8 mg of tissue removed at time of injury, males and females respectively), the number of myofibers in the mid cross-section (i.e., mid-belly of the muscle) was significantly reduced (interaction injury x whole muscle region, *p* = 0.001; Fig. 2C). Across the entire muscle, VML resulted in an ~ 45% and ~ 40% deficit in total myofiber number compared to injury Naïve and denervation, respectively. Within the mid cross-section of the gastrocnemius, neutral lipids (i.e., oil-red-o positive) were not affected by either injury (main effect of injury *p* = 0.758; Fig. 2D&F), although females had more neutral lipids than males (main effect of sex *p* = 0.014; Fig. 2F). Additional lipid species (i.e., BODIPY positive) were robustly apparent after VML. Specifically, there was a ~ 0.4% absolute difference in total lipid droplet positive area compared to Naïve and denervation (main effect of injury *p* = 0.001; Fig. 2E&G).

**Fig. 3:**
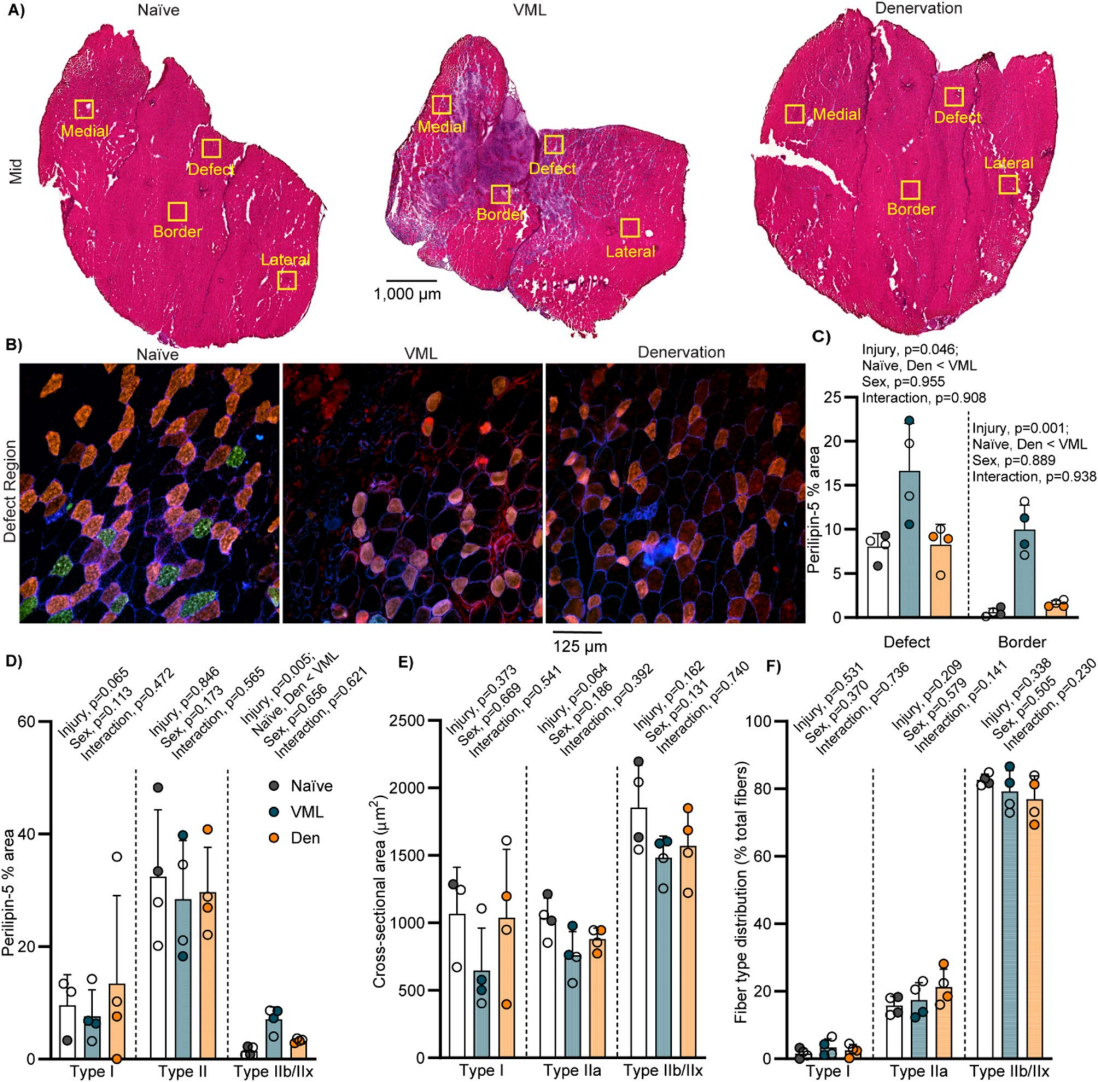
Fiber type-specific analysis of remaining muscle following injury. **A** Four regions of interests are noted on representative H&E-stained images of mid region of gastrocnemius muscle (see Fig. 5, images are replicated); scale bar is equal to 1,000 μm. **B** Representative images of the defect (or analogous location) stained for perilipin 5 (red), MyHC_slow_ (type I myofibers; green), MyHC_2A_ (type IIa myofibers; orange), and laminin (blue); scale bar is equal to 125 μm. **C** Percentage of perilipin 5 in defect and border regions of interest (main effect of injury *p* ≤ 0.046). **D** In a fiber-type specific approach, the area of perilipin 5 was greatest in type IIb/IIx myofibers (main effect of injury *p* = 0.005). The fiber type specific **E** cross-sectional area (*p* ≥ 0.064) and **F** distribution (*p* ≥ 0.141). Open circle female, closed male; dots represent an individual animal. Data for percent area of perilipin 5, cross-sectional area, and fiber type distribution were analyzed by two-way ANOVA; statistically significant Main Effects are noted; all p-values are shown within each graph and in Supplemental Table 1

Further characterization of MyHC isoform expression (i.e., muscle fiber types) and perilipin 5 expression in specific locations within the mid-belly of the muscle were done at standardized regions of interest in the VML defect and border (or analogous location), and a medial and lateral region distal from the injury location (Fig. 3A-B). Visualization and evaluation of the mid-belly of the muscle allows for the greatest contrast between remaining muscle, regenerating myofibers, and fibrotic or adipose infiltration [19] as well as being the location of the VML injury. At individual regions of interest, the area of perilipin 5 in the defect and border regions of VML injured muscle were ~ 2- and ~ 17-fold greater than Naïve, respectively (main effect of injury *p* ≤ 0.046; Fig. 3C). The average area of perilipin 5 across all regions of interest was significantly greater following VML only in type IIb/IIx myofibers (main effect of injury *p* = 0.005; Fig. 3D). The average cross-sectional area of all myofibers was 1486.7 ± 264.1µm^2^ (Fig. 3E). Neither sex nor injury impacted fiber type specific cross-sectional area and fiber type distribution (*p* ≥ 0.064; Fig. 3E-F). Type I myofibers exhibited an average cross-sectional area of 904.9 ± 414.8µm^2^ and accounted for an ~ 2.5% of all myofibers (Fig. 3E-F). Type IIa myofibers represented ~ 18% of all myofibers and had an average cross-sectional area of 892.9 ± 172.8µm^2^ (Fig. 3E-F). Lastly, average cross-sectional area of type IIb/IIx myofibers was 1636.2 ± 284.7µm^2^ and represented ~ 80% of all myofibers (Fig. 3E-F).

### Impact of injury on IGF-1

**Fig. 4:**
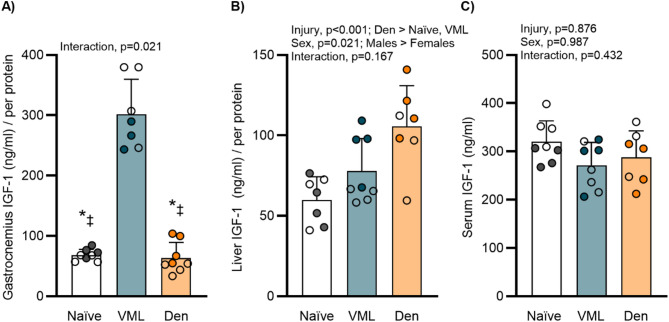
IGF-1 concentration across muscle, liver, and serum. **A** Normalized gastrocnemius muscle IGF-1 concentrations in the remaining muscle were greater in VML males and females compared to Naïve or denervation (interaction *p* = 0.021). **B** Normalized liver IGF-1 concentrations in liver (main effect of injury *p* < 0.001, sex *p* = 0.021) and **C** serum IGF-1 concentrations following injury (*p* ≥ 0.432). Open circle female, closed male; dots represent an individual animal. Data analyzed by two-way ANOVA; statistically significant Main Effects are noted; statistically significant Interactions are noted by: ‡ different than VML males, * different than VML females; all p-values are shown within each graph and in Supplemental Table 1

Insulin-like growth factor-1 (IGF-1) was examined in the remaining skeletal muscle, liver, and serum to characterize how injury impacts systemic and localized cellular environments that may have downstream effects on lipolysis. Males and females following VML injury exhibited (~ 3-fold) greater IGF-1 in the remaining gastrocnemius muscle compared to other groups (interaction *p* = 0.021; Fig. 4A). Denervation resulted in greater IGF-1 in the liver, with males across all groups exhibiting higher concentrations than females (main effect of injury *p* < 0.001, sex *p* = 0.021; Fig. 4B). There was no difference in circulating serum IGF-1 at 3 days post-injury across groups (*p* ≥ 0.432; Fig. 4C).

### Impact of injury on circulating and local adipokines

**Fig. 5:**
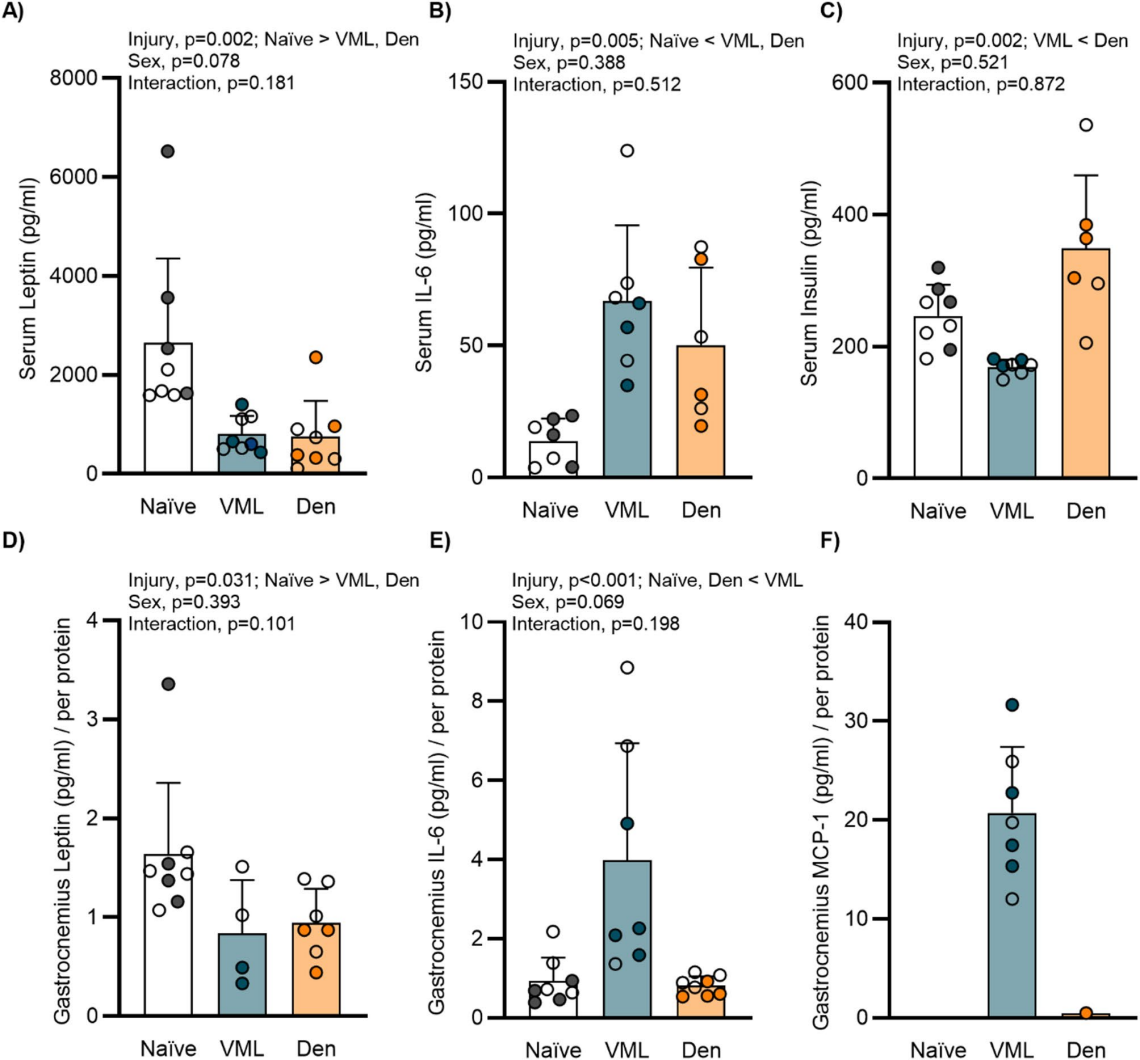
Adipokine concentrations across remaining muscle and serum following injury. **A** Serum leptin concentrations were blunted following injury (main effect of injury *p* = 0.002). **B** Serum IL-6 was greater following injury (main effect of injury *p* = 0.005). **C** Between injuries, serum insulin concentrations were lower following VML (main effect of injury *p* = 0.002). **D** Normalized leptin concentrations in the muscle remaining following injury were diminished compared to injury Naïve controls (main effect of injury *p* = 0.031). **E** Compared to other groups, VML exhibited greater IL-6 concentrations in the muscle remaining (main effect of injury *p* < 0.001). **F** MCP-1 was detectable following VML. Open circle female, closed male; dots represent an individual animal. Data analyzed by two-way ANOVA; statistically significant Main Effects are noted; all *p*-values are shown within each graph and in Supplemental Table 1

Circulating adipokines related to adipogenesis and metabolic regulation were evaluated to explore the adipokine response following injury. Both VML and denervation resulted in ~ 70% lower serum leptin than injury Naïve controls (main effect of injury *p* = 0.002; Fig. 5A). Conversely, serum IL-6 was greater in both VML and denervation compared to that of Naïve (main effect of injury *p* = 0.005; Fig. 5B). Though neither were distinct from injury Naïve controls, there was less serum insulin following VML injury compared to denervation (main effect of injury *p* = 0.002; Fig. 5C). Adipokines were also evaluated in the muscle remaining after injury, and like circulating levels, both injuries resulted in less leptin in the remaining muscle (main effect of injury *p* = 0.031; Fig. 5D). There was no difference in IL-6 between the denervated and Naïve muscle, however following VML, IL-6 was ~ 4-fold greater (main effect of injury *p* < 0.001; Fig. 5E). Monocyte chemoattractant protein-1 (MCP-1), a key factor that attracts and enhances inflammatory cells, was not detectable in the muscle of Naïve or denervated groups and did not meet a priori criteria for inclusion in our analyses. Of note, MCP-1 concentrations in the remaining muscle following VML were ~ 21 pg/ml per protein, likely due to excessive inflammation (Fig. 5F). Circulating adipokines and those in the remaining muscle may support lipid mobilization through an exacerbated inflammatory response, particularly following VML. Reduced signaling supportive of lipid storage (i.e., leptin, insulin) following VML injury may further contribute to lipid mobilization in the remaining muscle.

### Impact of injury on protein expression in the remaining muscle

**Fig. 6:**
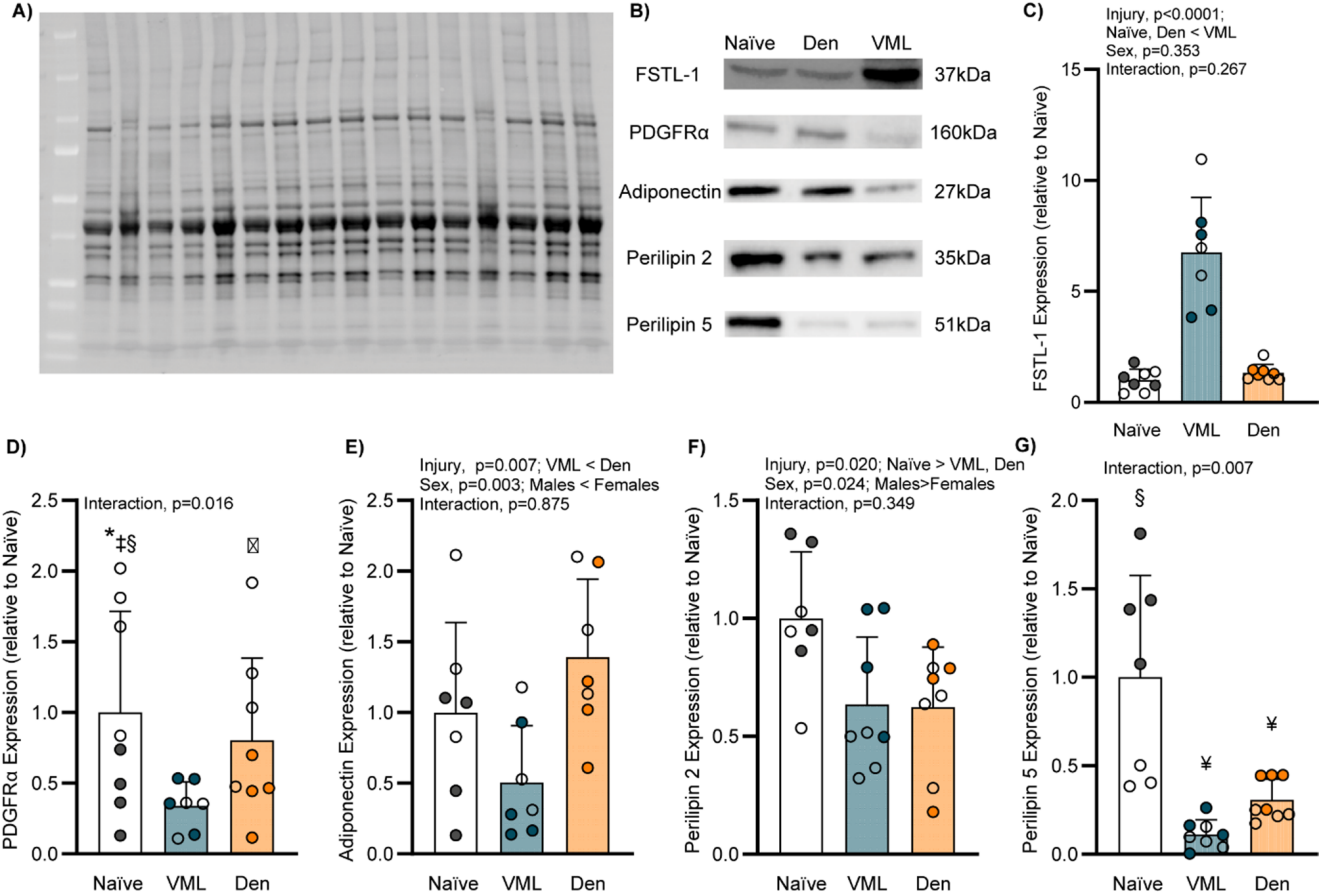
Protein expression in the remaining muscle following injury. Representative image of a **A** stain free blot for total lane protein quantification and **B** cropped chemiluminescence bands for quantification of protein expression. **C** VML exhibited greater FSTL-1 expression (main effect of injury *p* < 0.0001; sex *p* = 0.353). **D** Naïve females had greater PDGFRα expression compared to other groups (interaction *p* = 0.016). **E** VML resulted in decreased adiponectin expression compared to VML. Females had greater adiponectin expression than males (main effect of injury *p* = 0.007; sex *p* = 0.003). **F** Injury results in greater expression of perilipin 2, with males across all groups exhibiting greater expression than females (main effect of injury *p* = 0.020; sex *p* = 0.024). **G** Naïve and denervation females present with greater within-group perilipin 5 expression (interaction *p* = 0.007). Open circle female, closed male; dots represent an individual animal. Data analyzed by two-way ANOVA; statistically significant Main Effects are noted; statistically significant Interactions are noted by: ‡ different than VML males, * different than VML females, ɸ different than Naïve females, ¥ different than Naïve males, § sex differences within group; all *p*-values are shown within each graph and in Supplemental Table 1. Full-length blots for all proteins are presented in Supplementary Fig. 1

To assess whether the early post-injury muscle environment promotes lipid storage or mobilization, remaining muscle was analyzed for changes in lipid- and metabolism-associated markers. (Fig. 6A-B). Follistatin-like 1 protein (FSTL-1), which is implicated in promoting the differentiation of preadipocytes, is markedly increased following VML with a ~ 7-fold greater expression compared to Naïve (main effect of injury *p* < 0.0001; Fig. 6C). In contrast, the expression platelet-derived growth factor receptor alpha (PDGFRα) was blunted following VML with Naïve females exhibiting greater expression than other groups (interaction *p* = 0.016; Fig. 6D). While PDGFRα is typically linked to adipogenic differentiation, its decreased expression following VML may indicate changes in the regulation of lipids, potentially supporting lipolysis. Adiponectin was ~ 58% lower following VML compared to denervation injury (main effect of injury *p* = 0.007) with females exhibiting ~ 2-fold greater expression across groups (sex *p* = 0.003; Fig. 6E). Proteins associated with lipid metabolism, specifically the regulation of lipid droplets within the cell, are affected following both VML and denervation injury. Specifically, perilipin 2 was lower following both injuries (main effect of injury *p* = 0.020), with females exhibiting ~ 30% lower expression compared to males (main effect of sex *p* = 0.024; Fig. 6F). Perilipin 5 expression was different between sexes in both Naïve and denervation-injured muscles. These sex differences were not observed following VML, however, perilipin 5 expression following VML was ~ 92% and ~ 72% lower compared to Naïve and denervation males, respectively (interaction *p* = 0.007; Fig. 6G), positing that VML may affect the sex-specific response of perilipin 5. The protein expression in the remaining muscle following VML injury is suggestive of an environment that promotes lipid mobilization and may be part of the natural injury sequela.

## Discussion

Early characterization of the regulatory pathways and adipokine response offers valuable insight into how they may influence the development of metabolic diseases, cellular disruption and chronic functional impairments observed following both direct and indirect skeletal muscle injuries. Specifically following VML, alterations in whole-body and cellular lipid metabolism suggest that lipid droplets may play a role in the pathogenesis of skeletal muscle dysfunction. Identifying targets that exacerbate lipid droplet accumulation could aid in developing strategies to preserve long-term muscle functionality. Herein, the remaining muscle following VML exhibits an environment conducive to support lipid mobilization, which is further exacerbated by the known heightened inflammatory response. Lipid droplet-associated proteins (e.g., perilipin 5) are localized within skeletal muscle regions near the initial VML injury, with greater accumulation in type IIb/IIx myofibers. Moreover, disruptions in adipogenic regulation and lipid droplet accumulation are unique to the non-recoverable skeletal muscle injury (i.e., VML) and occur as early as three days post-injury. Adipokines associated with adverse whole-body metabolic disruption (e.g., adiponectin) are evident particularly following VML. Early changes within the muscle environment may limit the functional capacity and metabolism of both injuries chronically. Examining how lipid mobilization may be acutely influenced following skeletal muscle injuries may reveal targets for early intervention and mitigation of long-term muscle and metabolic dysfunction.

Both VML and denervation exhibit immediate functional deficits three days post-injury. It is expected that even though there was no muscle function at three days following denervation, recapitulation of function can occur given significant time and/or intervention [48–51]. The non-recoverable functional deficits following VML have been well established, and are reinforced here, even at an early time-point [19, 29, 41]. The functional impact at the myofiber directly also varies across these two traumatic conditions. With the initial direct loss of myofibers at the time of VML, there is an overall decrease in whole muscle size and myofiber number, but the myofibers remaining are not found to be atrophic [28, 52]. In contrast, denervation causes long-term impacts on whole muscle size resultant to atrophy of individual myofibers. Given opposing functional trajectories of denervation and VML injuries, comparing these two models provides insight into the immediate histological and biochemical impacts of the injuries. These injury models are standardized and attempt to translate to clinical injuries, it is acknowledged that injuries involving denervation and VML are multifaceted and include multiple mechanisms of injury.

Perilipin 2, which is primarily responsible for protecting skeletal muscle lipid droplet stores and preventing lipid mobilization [53, 54], is blunted following VML. Though not directly involved in facilitating fatty acid oxidation, perilipin 2 inhibits adipose triglyceride lipase (ATGL) at the lipid droplet surface, thus preventing lipolysis [55]. Three days post-injury, the low expression of perilipin 2 suggests an immediate metabolic shift to promote substrate (i.e., fatty acids) availability and prioritize fatty acid oxidation. The low circulating insulin levels observed post-VML herein could further promote lipolysis and lipid mobilization. Supported by a modest, yet significant, accumulation of neutral lipids (i.e., BODIPY-positive) in the remaining muscle following VML, which likely reflects an increase in lipid mobilization. Recently the C57BL/6J rodent strain was noted to be more resistant to lipid accumulation following a localized skeletal muscle injury than other strains, which may contribute to the reported modest accumulation here [56]. Comparatively, localized skeletal muscle models of injury such as intramuscular cardiotoxin or glycerol injections, result in known alterations in lipid signaling and accumulation; the accumulation has been reported to impair skeletal muscle function following injury [57, 58]. Notably, the location of lipids in either the extracellular matrix or intramyocellular space is thought to influence functional outcomes [59]. Characterizing the location of lipid accumulation post-VML could provide insight into how lipids may influence the functional deficits that occur. While injection models could be an adequate model to understand mechanisms of muscle regeneration, they are insufficient as translatable models for clinical conditions, thus highlighting novelty of exploring this phenomenon in VML injury and denervation models.

Skeletal muscle pathologies associated with chronic muscle wasting and functional deficits (i.e., sarcopenia and cachexia) are associated with increases in perilipin 2 expression [60, 61]. In these pathologies, perilipin 2 is implicated in the prevention of lipotoxicity from chronic stress and inflammation [62]. Furthermore, lipid droplet Content within skeletal muscle correlates with perilipin 2 expression, suggesting that perilipin 2 may contribute to excessive lipid accumulation and muscle dysfunction. Previously reported temporal impairments in mitochondrial respiration and conductance following VML occur independent of free fatty acid availability [27]. Though low perilipin 2 expression at three days post-injury may support an increase in lipolysis, known mitochondrial dysfunction following VML may impair the effective utilization of fatty acid substrates and limit the ability to meet metabolic demands at the cellular level. The long-term trajectory of perilipin 2 following VML and denervation remains unclear, but its failure to be properly regulated may lead to uncontrolled lipid mobilization and further functional impairments.

Lipid droplet-associated proteins show distinct patterns that vary depending on myofiber type and the location within the muscle, specifically following VML. Perilipin 5, the protein primarily responsible for facilitating the physical connection between lipid droplets to mitochondria [63], is abundant in the VML defect and bordering region of the injury. Herein, perilipin 5 was evaluated by quantifying protein expression and its histological distribution in the defect-containing portion of the gastrocnemius muscle. Increases in perilipin 5 protein expression suggest greater oxidative capacity and mitochondrial energy efficiency [64], with robust histological expression in type I myofibers [65, 66]. Three days post-VML, the area of perilipin 5 observed within the VML defect and bordering regions was increased. In the proximal portion of the gastrocnemius, protein expression of perilipin 5 was reduced post-VML, suggesting a region-specific regulation of perilipin 5 that may reflect localized metabolic adaptation. This difference may also be due to the regulatory role of perilipin 5. Specifically, PKA-mediated phosphorylation causes perilipin 5 to detach from lipid droplets and translocate to the nucleus, there it helps initiate the transcription of mitochondrial biogenesis-related genes [30]. It is possible that protein expression quantification in the muscle may not be capturing this phenomenon. Additionally, an increased histological area of perilipin 5, particularly near the VML injury site, may represent a spatially relevant response to promote fatty acid oxidation, acutely. Pathological accumulation of lipids and lipid droplet-associated proteins are reported across metabolic diseases (e.g., type 2 diabetes), and accumulation is robust in type II myofibers [67, 68]. Three days following VML, the area of perilipin 5 is greater in type IIb/IIx fibers, indicating a fiber-specific alteration in perilipin 5 that resembles changes in chronic metabolic diseases, though the small sample size of our histology cohort may limit the ability to draw definitive conclusions about the relevance of this finding. It is unknown whether the decrease in perilipin 2 and increase in perilipin 5 post-VML is resultant to an increase in metabolic demands for muscle repair of the remaining muscle. The accumulation of lipids and lipid droplet-associated proteins three days post-injury could signal the beginning of a pathological response.

Adipokines, which regulate energy balance and metabolic health, are disrupted in the remaining muscle following VML. At three days post-VML, the remaining muscle has reduced adiponectin expression and increased pro-inflammatory adipokines. Though denervation also presents with an increase in pro-inflammatory adipokines, adiponectin remains unchanged. An exacerbated pro-inflammatory (e.g., CD8^+^, CD45^+^) state has been demonstrated post-VML and is implicated in contributing to failed regeneration [69, 70]. Marked increases in IL-6 at three days post-injury demonstrated herein may be contributing to lower adiponectin expression. Chronic increases in adiponectin in the remaining muscle post-VML are observed alongside whole-body metabolic impairments [18]. Specifically, decreases in whole-body lipid oxidation and diurnal impairments in metabolic flexibility have been reported following the injury [19, 20]. Adiponectin has also been implicated to the modulation of lipid metabolism and contributing to metabolic flexibility [71]. Suggesting adiponectin may be an appropriate marker post-VML that occurs concomitantly with whole-body metabolic impairments. Low adiponectin is often associated with skeletal muscle pathologies that exhibit chronic systemic inflammation (e.g., muscle wasting) and impaired functional capacity. Similarly, leptin levels can influence skeletal muscle by facilitating increases in muscle mass and functional properties [72]. Adiponectin and leptin serve as key markers for overall metabolic health, and dysregulation three days post-injury may be an indicator of metabolic imbalance. The data herein demonstrate that immediate adipokine disruption occurs following VML. Further work should examine these adipokines across the time course of an injury, particularly a direct and non-recoverable injury such as VML.

## Conclusions

Early disruptions in lipid metabolism and adipokine signaling following direct and indirect muscle injuries provide insights into the mechanisms driving chronic functional and metabolic impairments. Distinct expression of lipid droplet-associated proteins occurring in the remaining muscle following VML indicate an early metabolic shift that may predispose remaining muscle to lipid perturbations. Moreover, the dysregulation of adipokines underscores a localized environment conditioned for chronic inflammation and impaired metabolic flexibility. Given late-stage lipid accumulation following VML has been largely anecdotally noted [73] and limitedly analyzed [74], these results are the first to quantitively report early-stage lipid accumulation following VML. These early, injury-specific changes, particularly in a direct and non-recoverable injury, may underpin why individuals undergoing limb salvage develop cardiometabolic comorbidities (i.e., obesity and hyperlipidemia) at rates comparable to those with limb amputation, despite preservation of the limb [75]. While limb salvage aims to protect aspects of mobility and promote eventual recapitulation of limb function [76], it remains unclear if early lipid accumulation following direct traumatic injury is detrimental to late-stage tissue remodeling and functionality. Thus, understanding whether these early metabolic and inflammatory disruptions represent an acute response or mark the beginning of a progressive trajectory is essential for the development of subsequent interventions and mitigation of adverse lipid accumulation.

## Supplementary Information

Supplementary Material 1.

Supplementary Material 2.

## Data Availability

The datasets used and/or analyzed during the current study are primarily presented in the current manuscript and are available from the corresponding author on request.

## Abbreviations

FSTL-1: Follistatin-like 1 protein
IGF-1: Insulin-like growth factor-1
IL-6: Interleukin-6
MCP-1: Monocyte chemoattractant protein-1
MyHC: Myosin heavy chain
PDGFRα: Platelet-derived growth factor alpha
VML: Volumetric muscle loss
