# Supplementary material for: Accumulation of lipids after acute direct and indirect traumatic injuries in male and female mice
Source: BMC Musculoskelet Disord. 2025 Oct 8;26:932. doi: 10.1186/s12891-025-09207-5 (PMC12505983; doi:10.1186/s12891-025-09207-5)
Supplement: Supplementary file 1 — Supplementary Material 1. [file 12891_2025_9207_MOESM1_ESM.pdf]

Supplemental Table 1. Statistical results of variables

		2 Way ANOVAs		
**Variables**	**Figure**	**Main effect**		**Interaction**
		**Injury**	**Sex**	**Injury*Sex**
Gastroc mass : body mass	1A	**<0.0001**	0.352	0.965
Maximal isometric torque	1B	**<0.0001**	0.690	0.906
Fatique index	1C	**0.016**	0.245	0.338
Passive torque at 20* dorsiflexion	1D	**0.001**	**<0.0001**	0.331
Oil-Red-O positive area	2F	0.758	**0.014**	0.379
BODIPY positive area	2G	**0.001**	0.330	0.840
% area Perilipin 5 defect region	3C	**0.046**	0.955	0.908
% area Perilipin 5 border region	3C	**0.001**	0.889	0.938
% area Perilipin 5 Type I	3D	0.065	0.113	0.472
% area Perilipin 5 Type IIa	3D	0.846	0.173	0.565
% area Perilipin 5 Type IIb/IIx	3D	**0.005**	0.656	0.621
Cross sectional area Type I	3E	0.373	0.669	0.541
Cross sectional area Type IIa	3E	0.064	0.186	0.392
Cross sectional area Type IIb/IIx	3E	0.162	0.131	0.740
Fiber type distribution Type I	3F	0.531	0.370	0.736
Fiber type distribution Type IIa	3F	0.209	0.579	0.141
Fiber type distribution Type IIb/IIx	3F	0.338	0.505	0.230
Gastrocnemius IGF-1	4A	-	-	**0.021**
Liver IGF-1	4B	**<0.001**	**0.021**	0.167
Serum IGF-1	4C	0.876	0.987	0.432
Serum Leptin	5A	**0.002**	0.078	0.181
Serum IL-6	5B	**0.005**	0.388	0.512
Serum Insulin	5C	**0.002**	0.521	0.872
Gastrocnemius Leptin	5D	**0.031**	0.393	0.101
Gastrocnemius IL-6	5E	**<0.001**	0.069	0.198
Gastrocnemius MCP1	5F	-	-	-
FSTL-1 protein expression	6C	**<0.0001**	0.353	0.267
PDGFRa protein expression	6D	-	-	**0.016**
Adiponectin protein expression	6E	**0.007**	**0.003**	0.875
PLPN2 protein expression	6F	**0.020**	**0.024**	0.349
PLPN5 protein expression	6G		-	**0.007**

		3 Way ANOVAs						
		**Main effect**			**Interaction**			
		**Injury**	**Sex**	**Whole Muscle Region**	**Injury*Sex**	**Injury*Whole Muscle Region**	**Sex*Whole Muscle Region**	**Injury*Sex* Whole Muscle Region**
Myofiber Number	2C	**<0.001**	**0.031**	**<0.0001**	0.183	**0.001**	0.882	0.603
