# Supplementary material for: Accumulation of lipids after acute direct and indirect traumatic injuries in male and female mice
Source: BMC Musculoskelet Disord. 2025 Oct 8;26:932. doi: 10.1186/s12891-025-09207-5 (PMC12505983; doi:10.1186/s12891-025-09207-5)
Supplement: Supplementary file 2 — Supplementary Material 2. [file 12891_2025_9207_MOESM2_ESM.pdf]

Accumulation of lipids after acute direct and indirect traumatic injuries in male and female mice

Angela S. Bruzina,[1] Braydon A. Crum,[1] Christiana J. Raymond-Pope,[1] Jarrod A. Call[2,3] & Sarah M. Greising[1]*

[1]School of Kinesiology, University of Minnesota, Minneapolis MN 55455, USA

[2]Department of Physiology and Pharmacology, University of Georgia, Athens, GA 30602, USA

[3]Regenerative Bioscience Center, University of Georgia, Athens, GA 30602, USA

Supplemental Figure 1

Follistatin-like protein (FSTL-1)
Stain free gel 1
Figure 6C

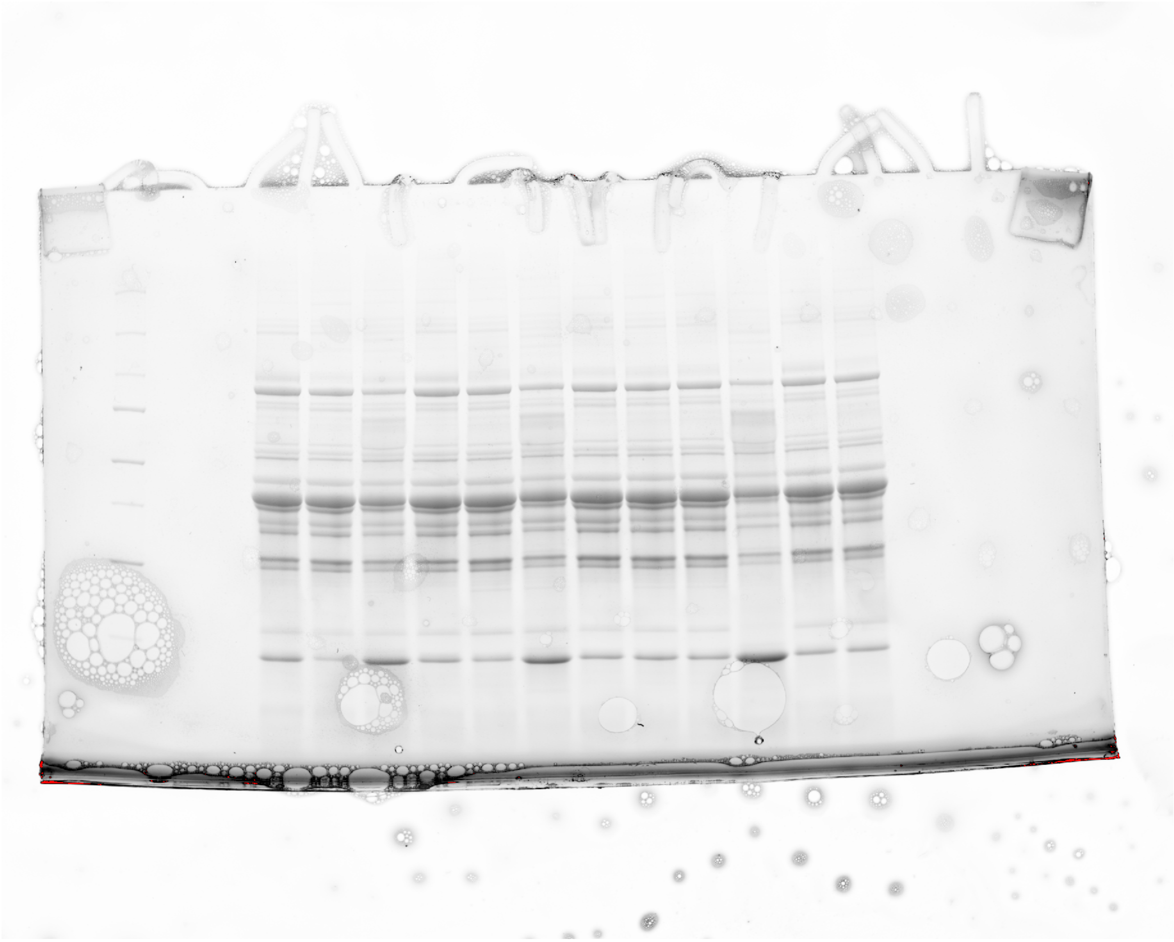

Follistatin-like protein (FSTL-1)
Stain free blot 1
Figure 6C

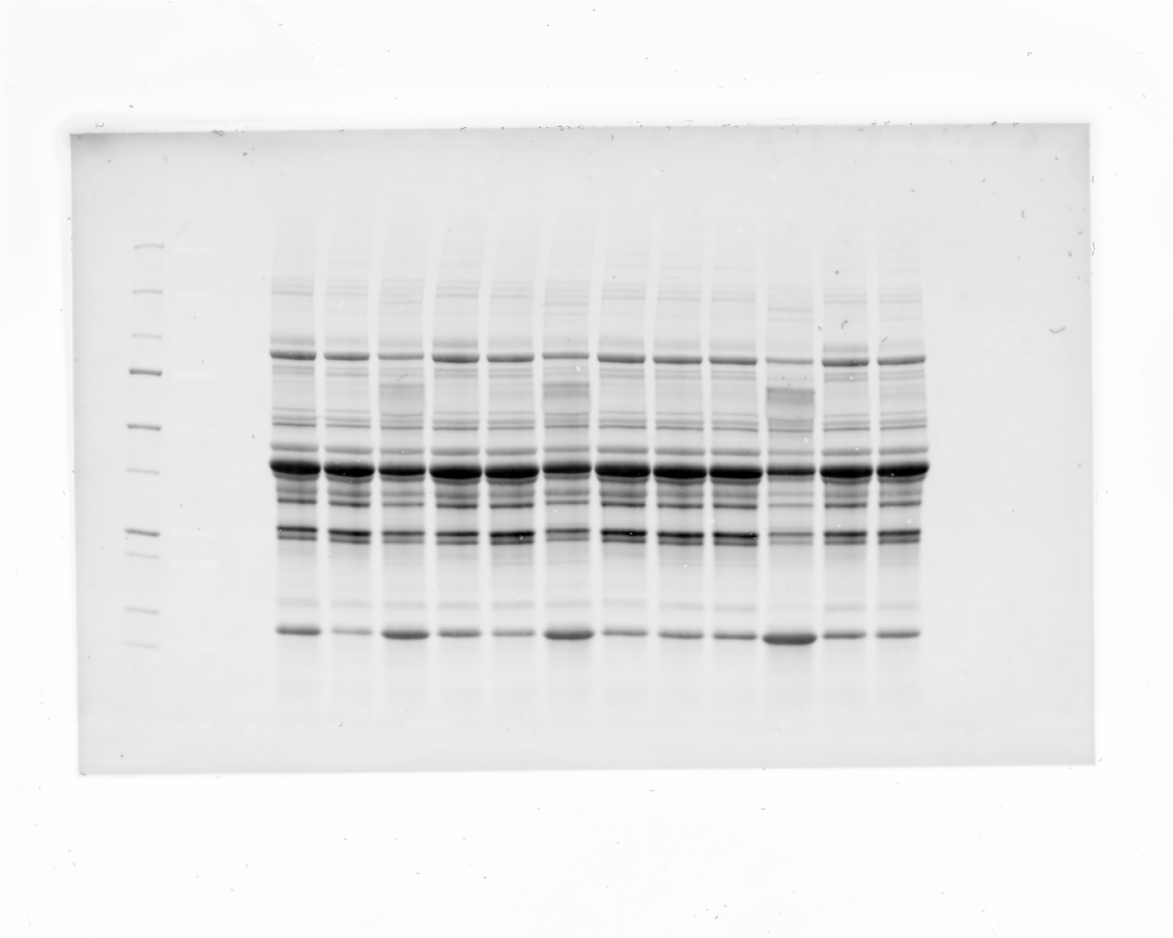

Follistatin-like protein (FSTL-1)
Chemiluminescence blot 1
Figure 6C

Representative images and groups are marked with red box

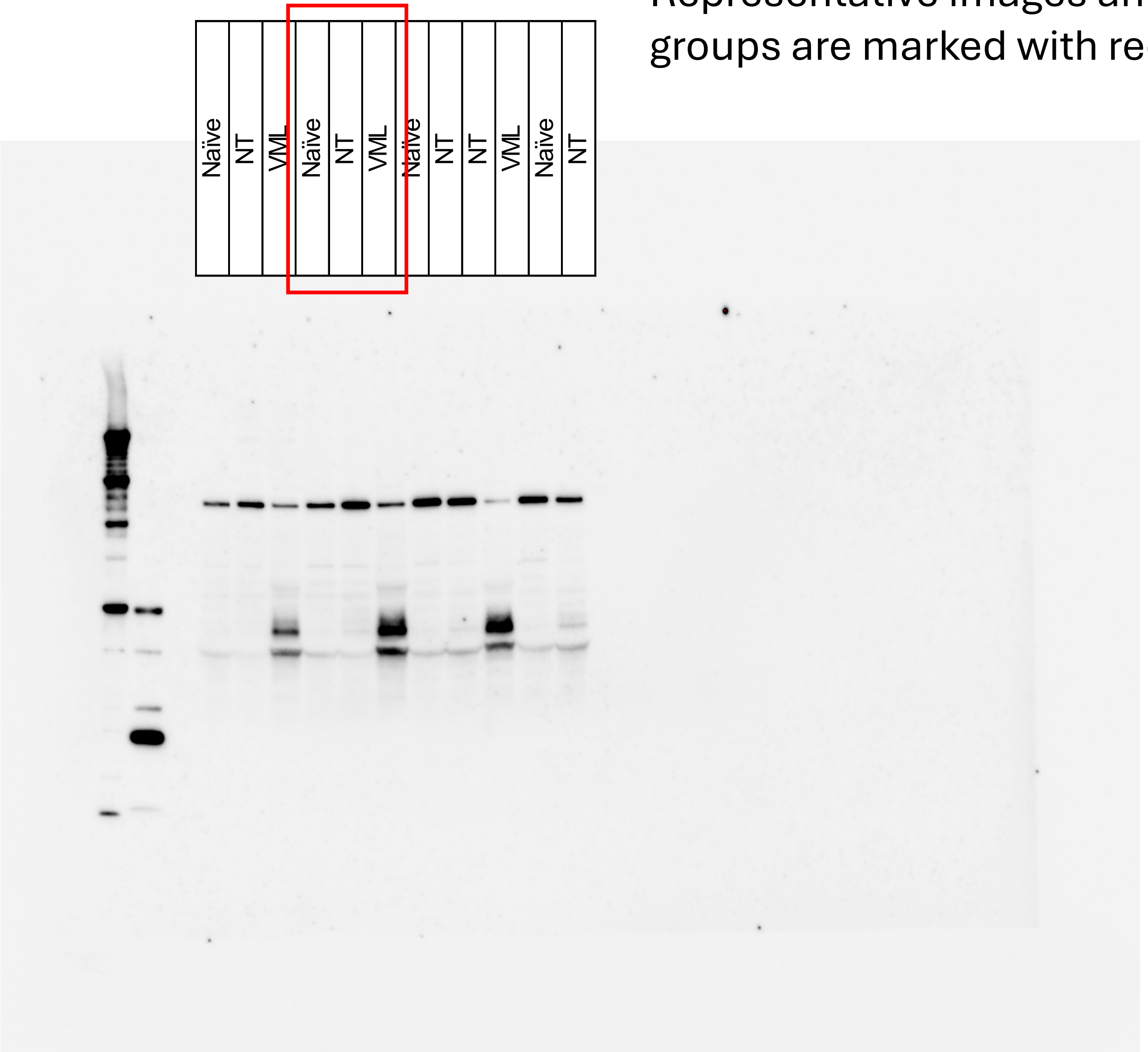

Follistatin-like protein (FSTL-1)
Stain free gel 2
Figure 6C

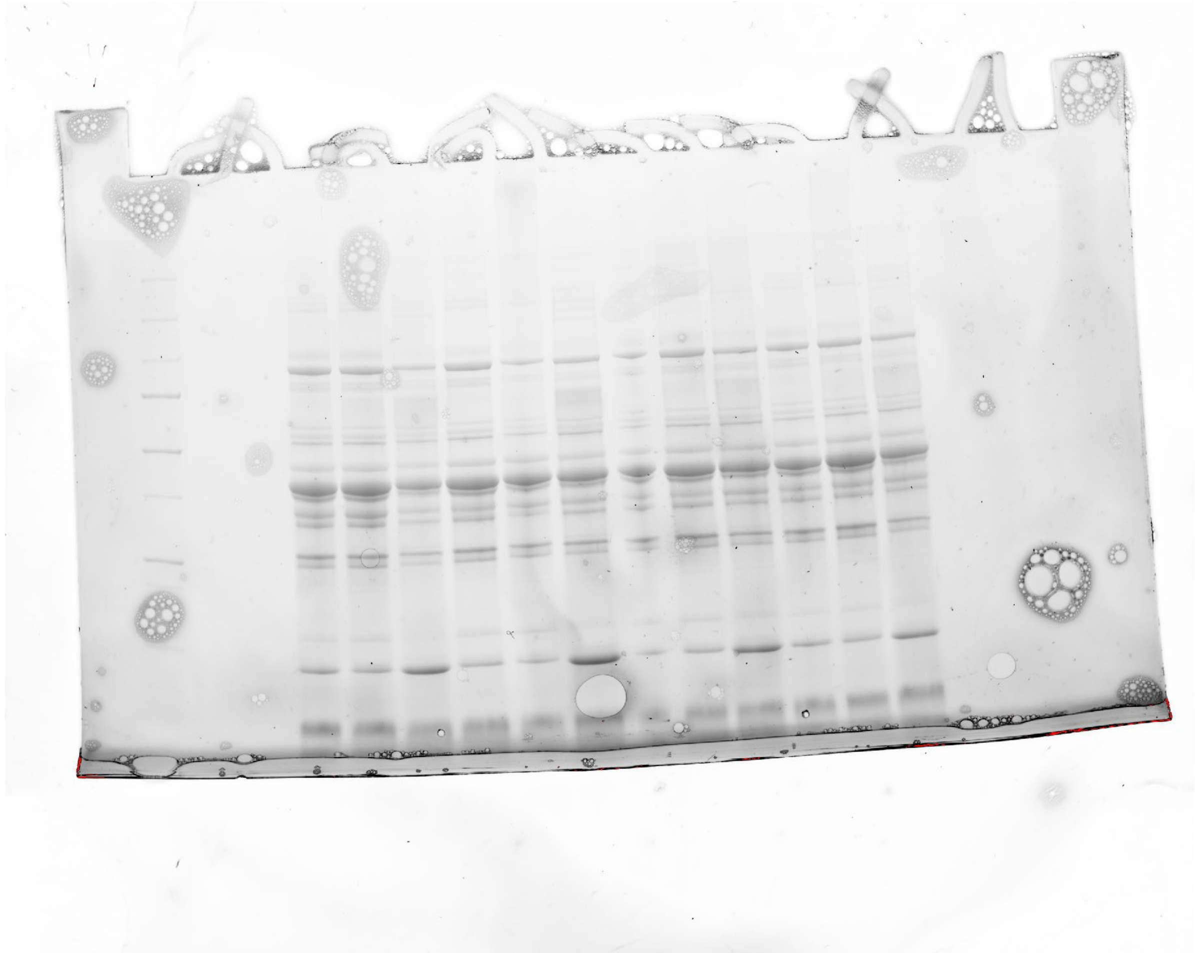

Follistatin-like protein (FSTL-1)
Stain free blot 2
Figure 6C

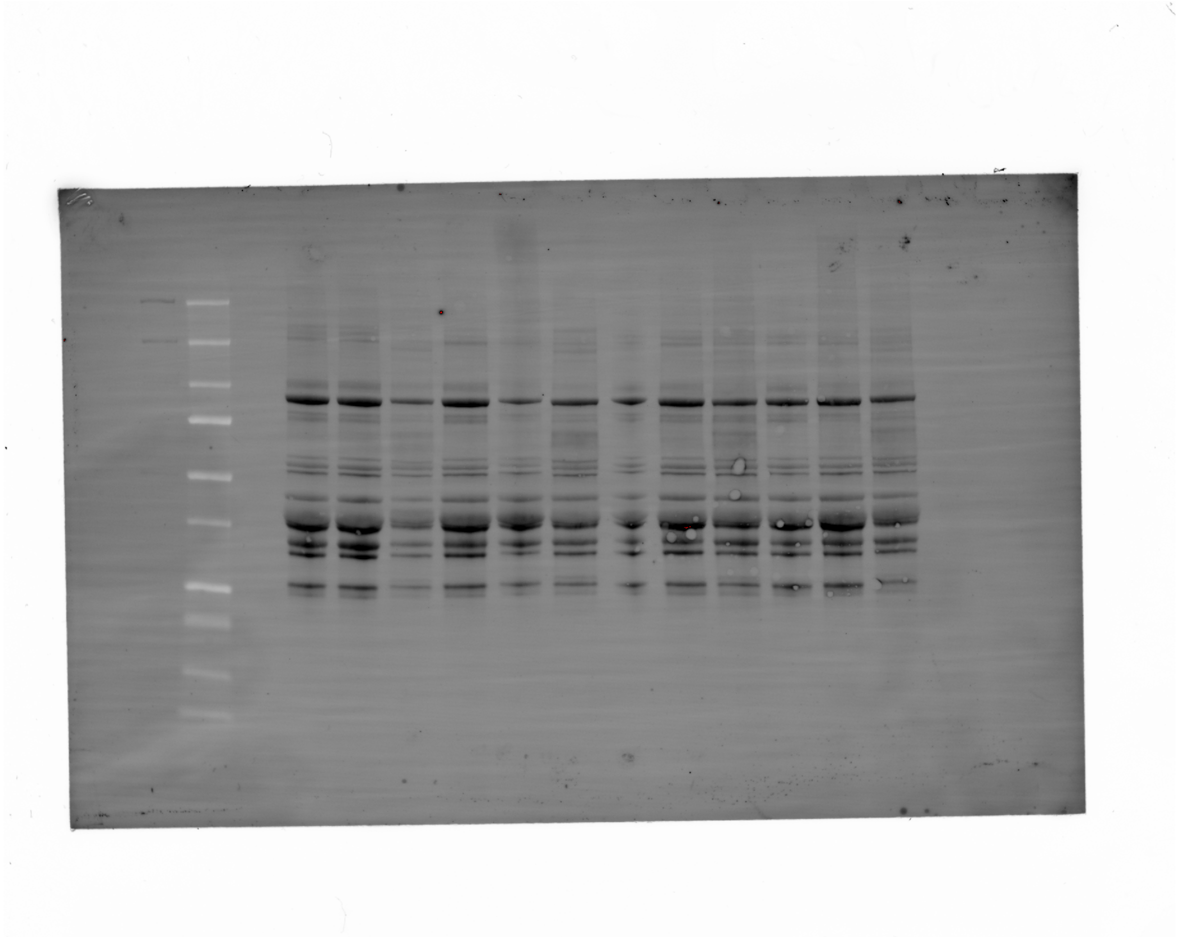

Follistatin-like protein (FSTL-1)
Chemiluminescence blot 2
Figure 6C

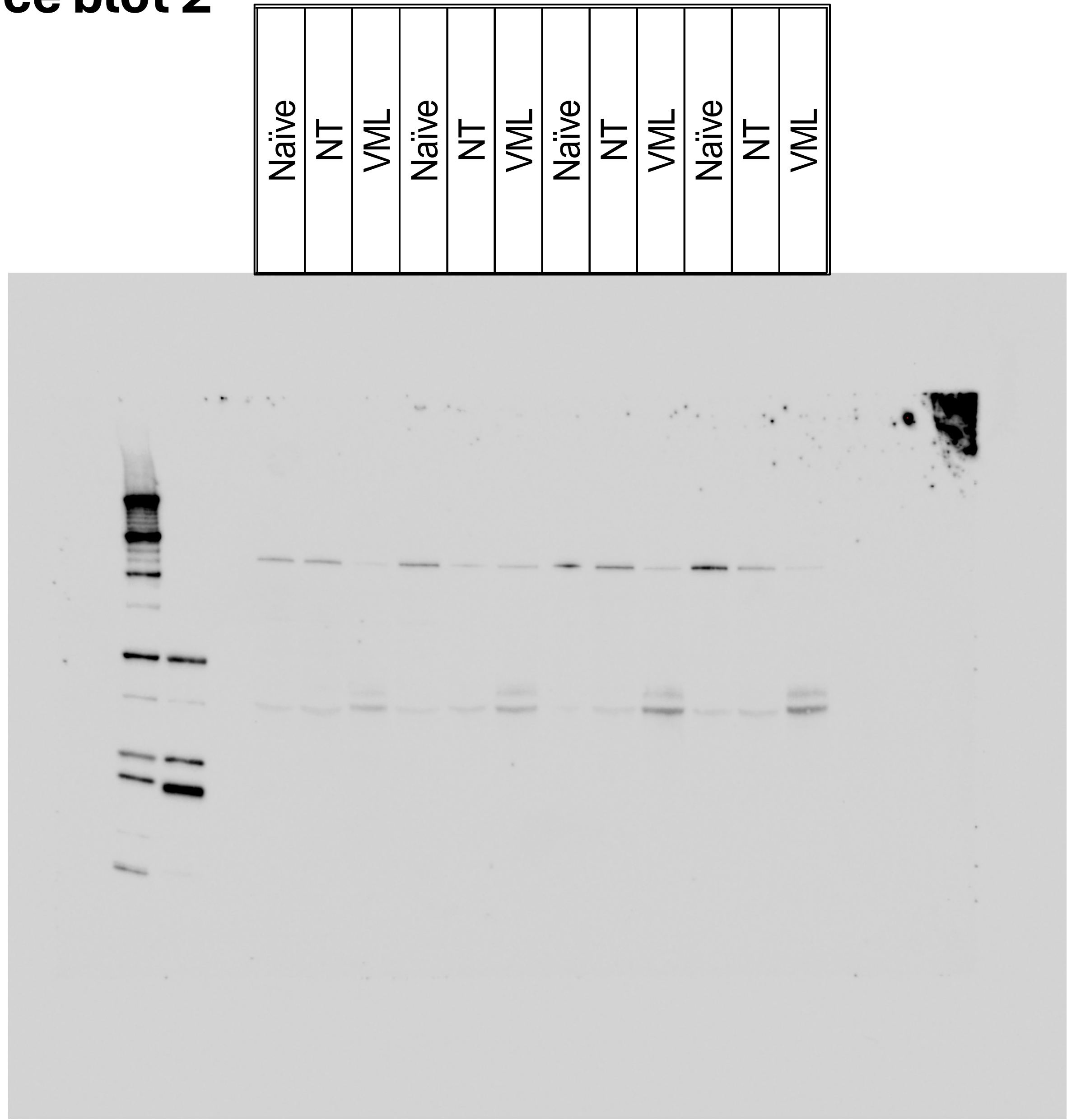

Platelet-derived growth factor alpha (PDGFRα)
Stain free gel 1
Figure 6D

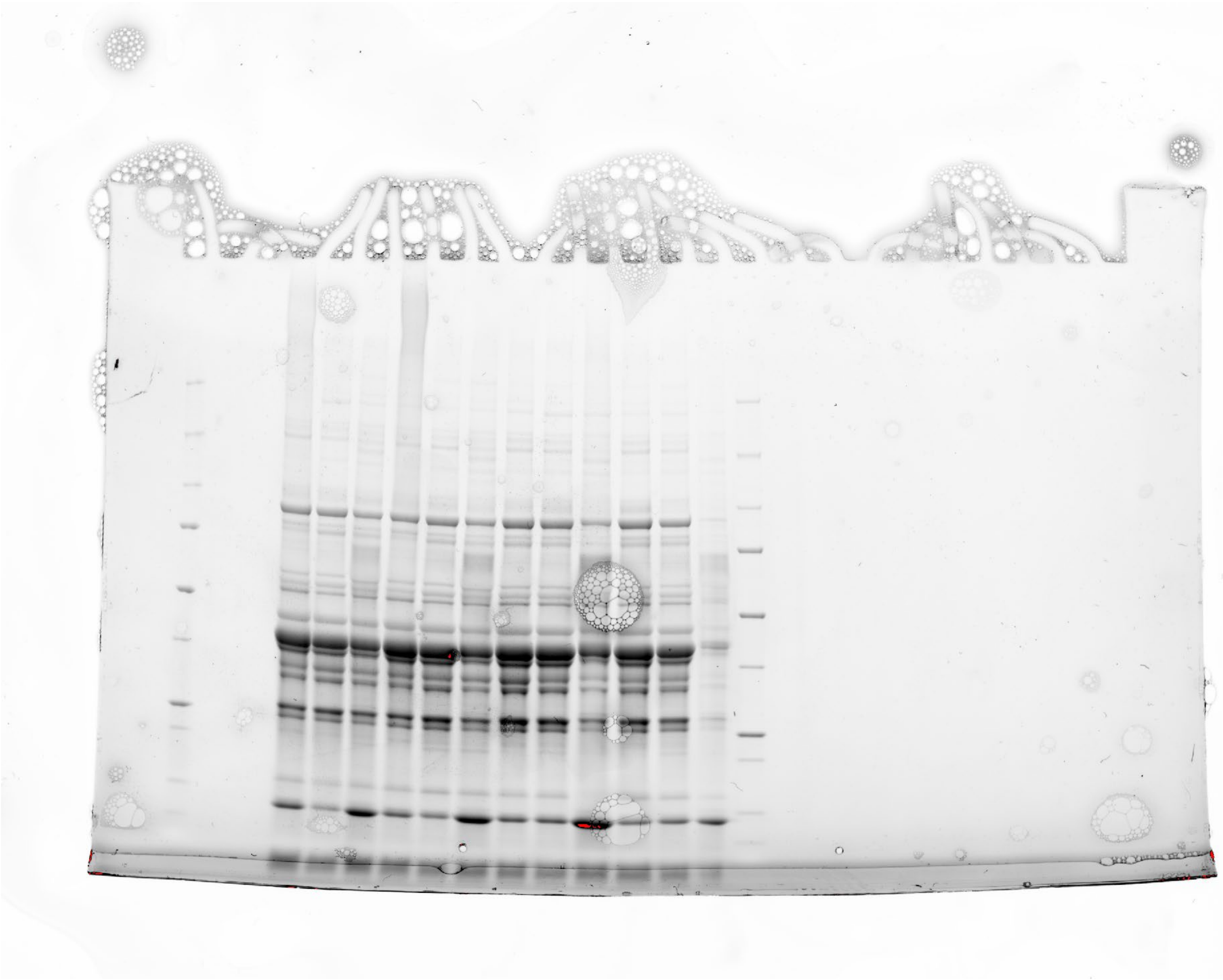

Platelet-derived growth factor alpha (PDGFRα)
Stain free blot 1
Figure 6D

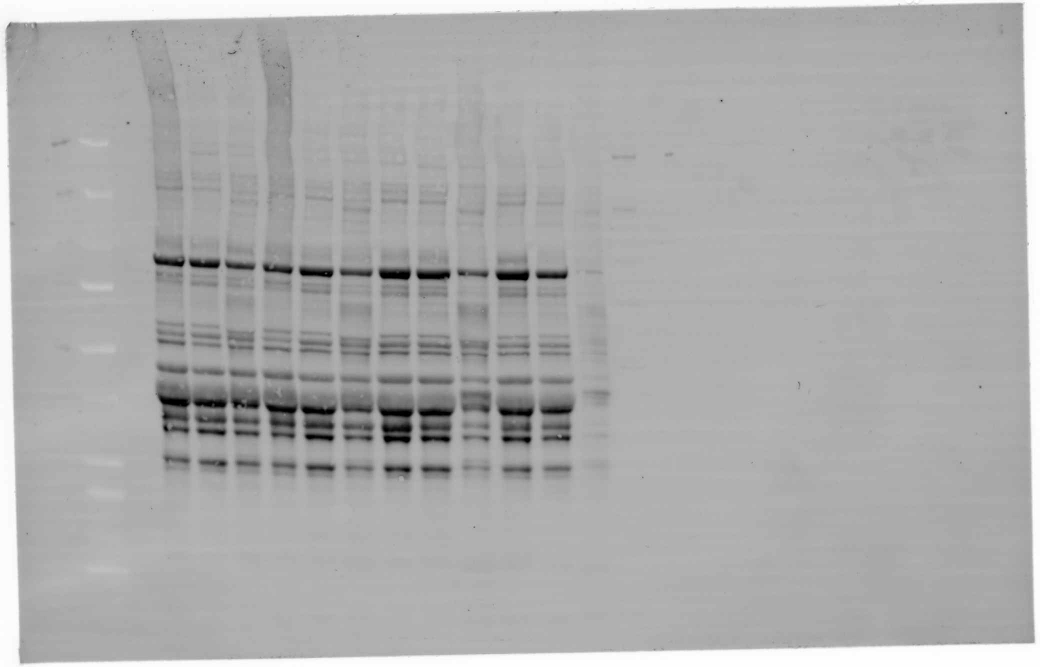

Platelet-derived growth factor alpha (PDGFRα)
Chemiluminescence blot 1
Figure 6D

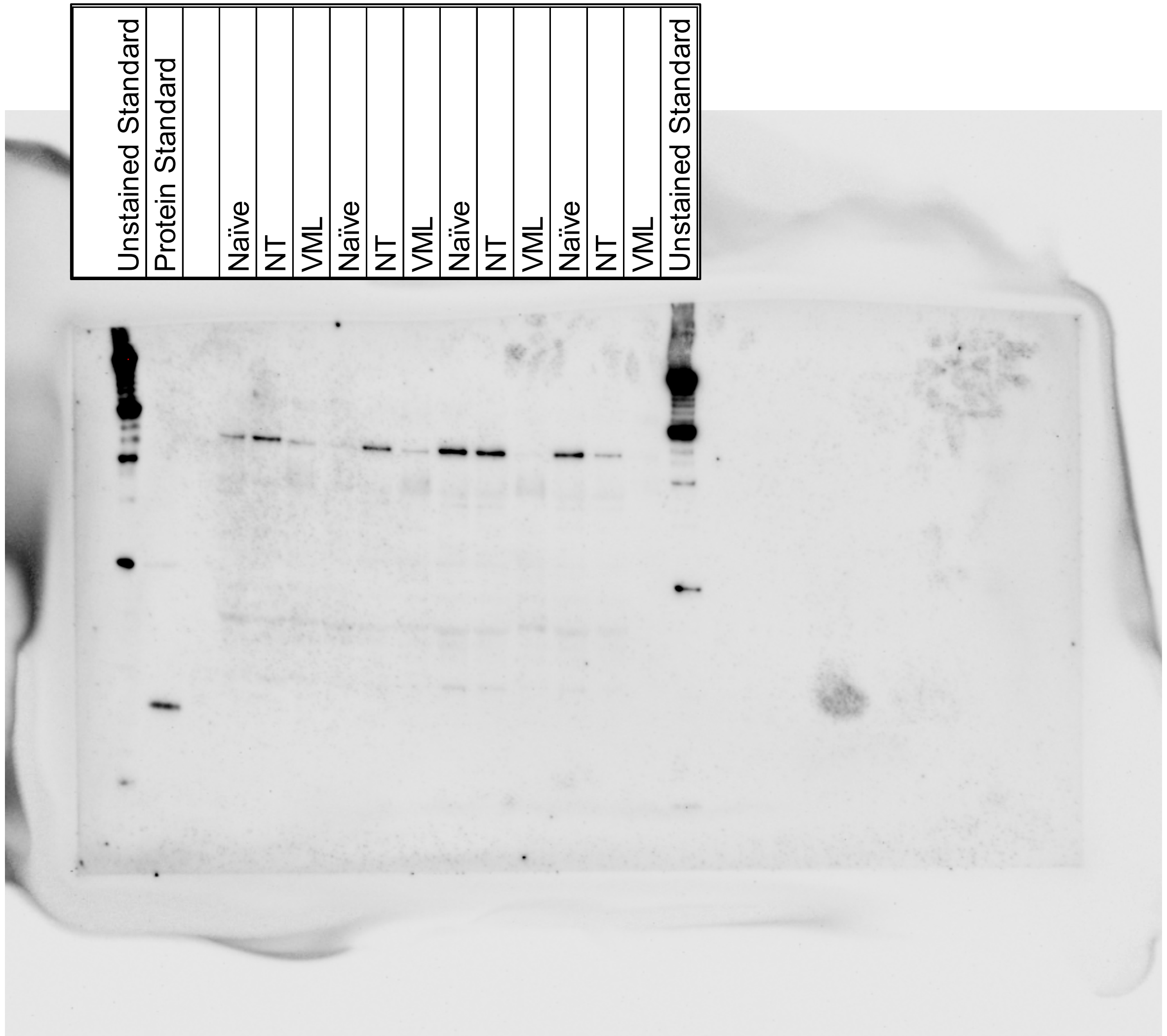

Platelet-derived growth factor alpha (PDGFRα)
Stain free gel 1
Figure 6D

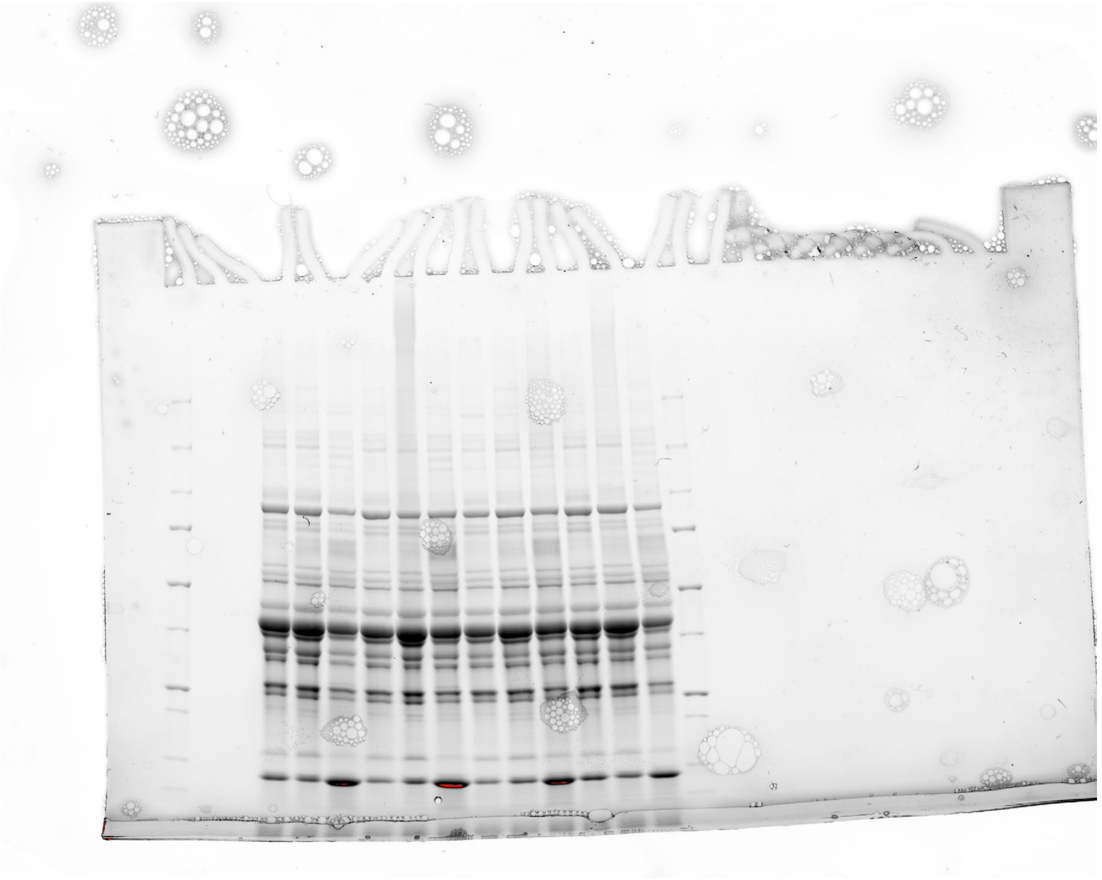

Platelet-derived growth factor alpha (PDGFRα)
Stain free blot 1
Figure 6D

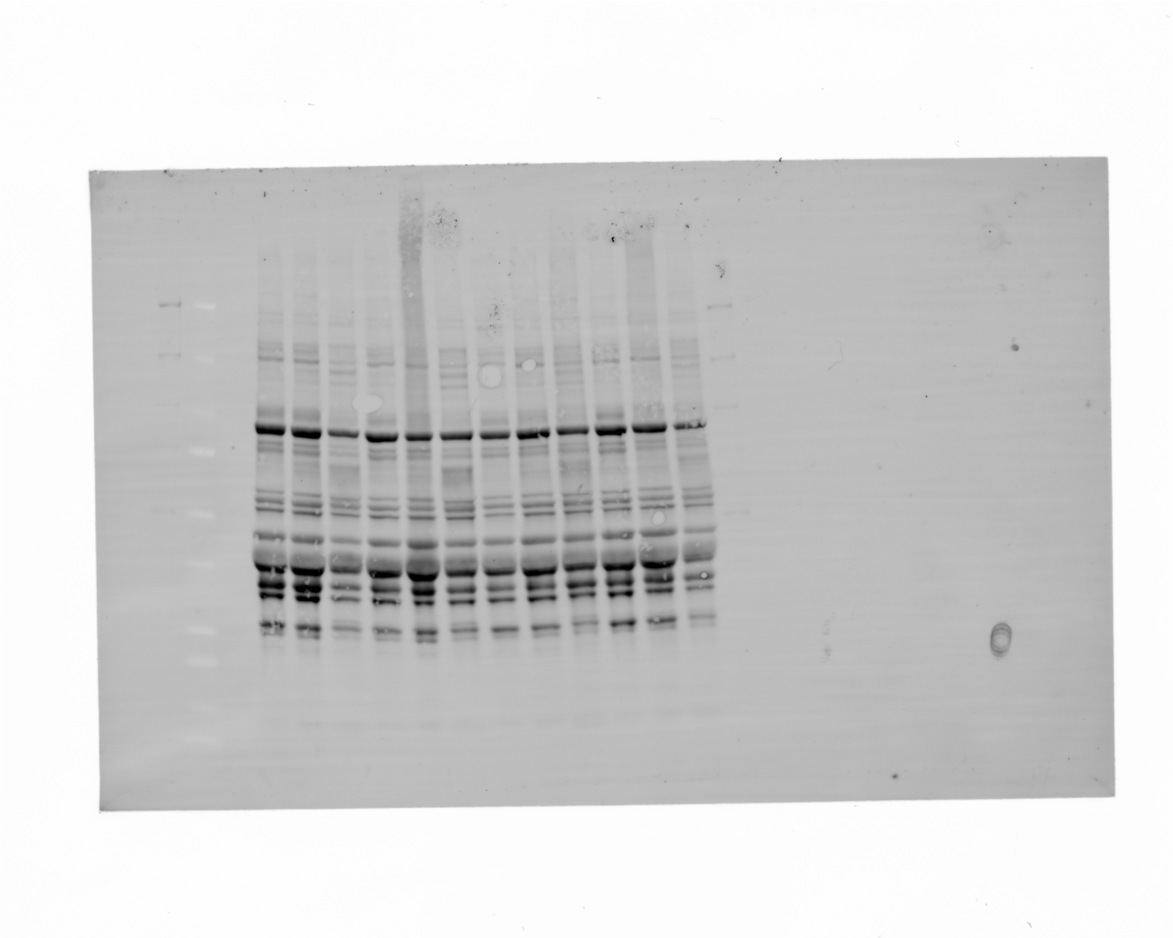

Platelet-derived growth factor alpha (PDGFRα)
Chemiluminescence blot 2
Figure 6D

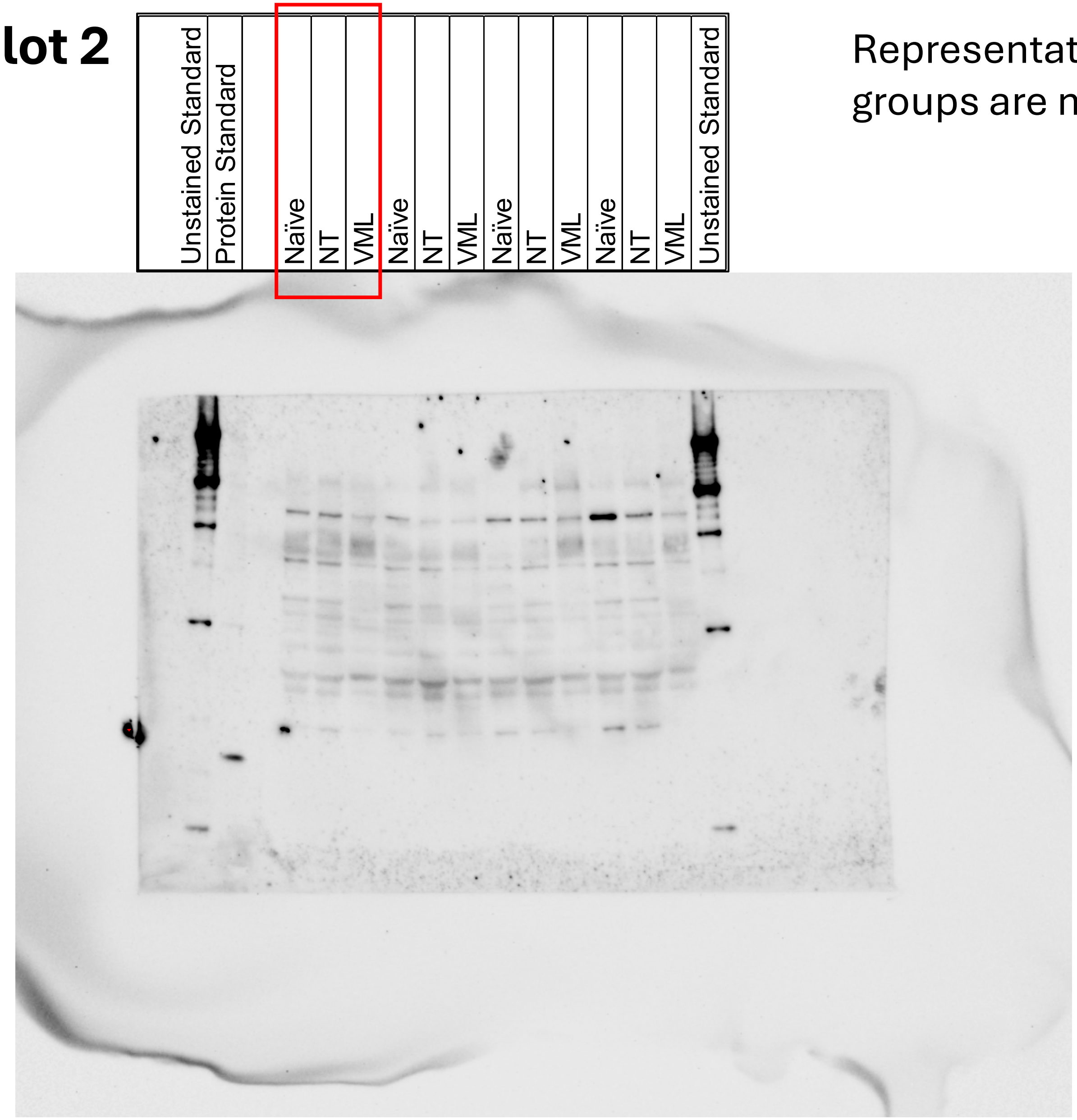

Representative images and groups are marked with red box

Adiponectin
Stain free gel 1
Figure 6E

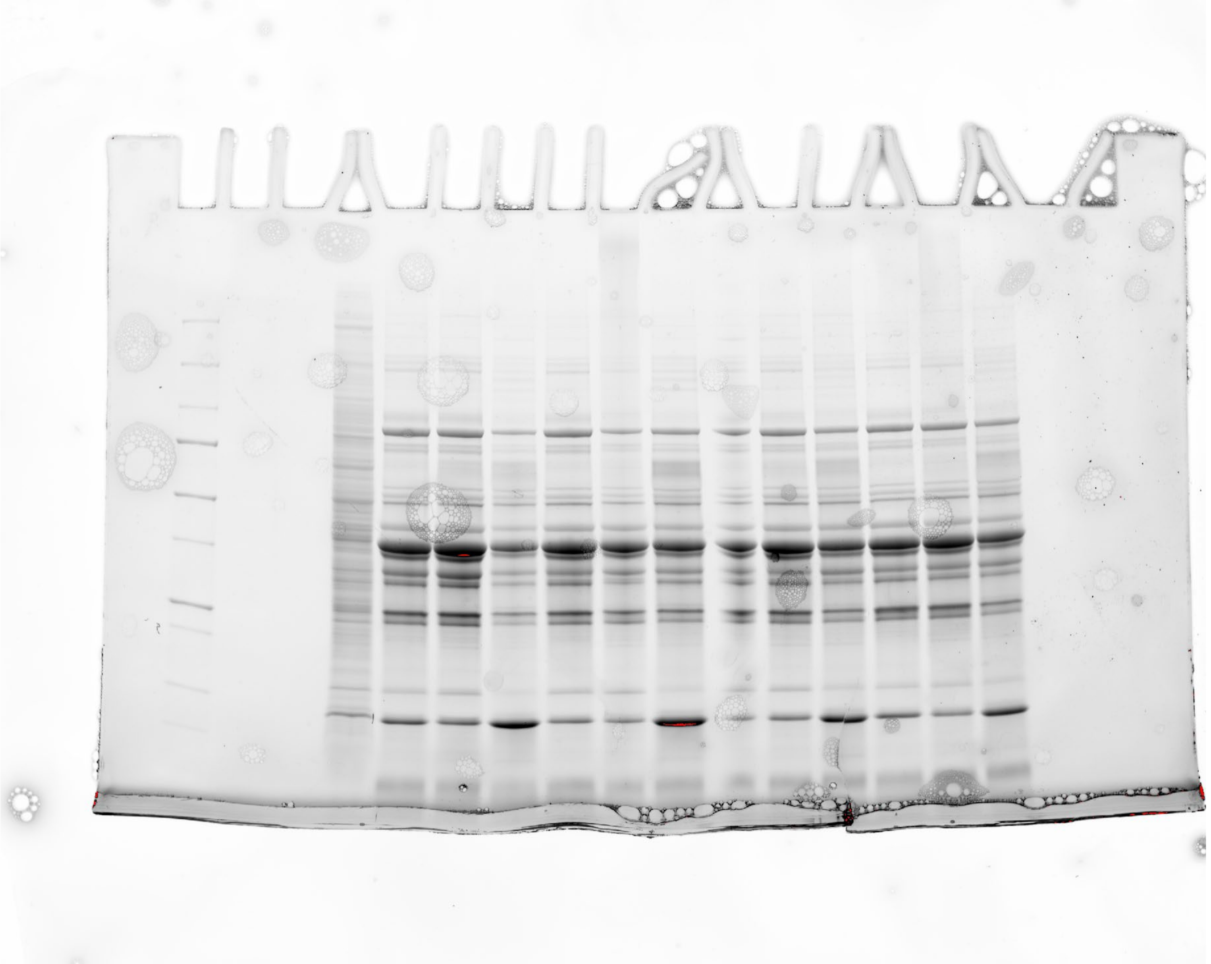

Adiponectin
Stain free blot 1
Figure 6E

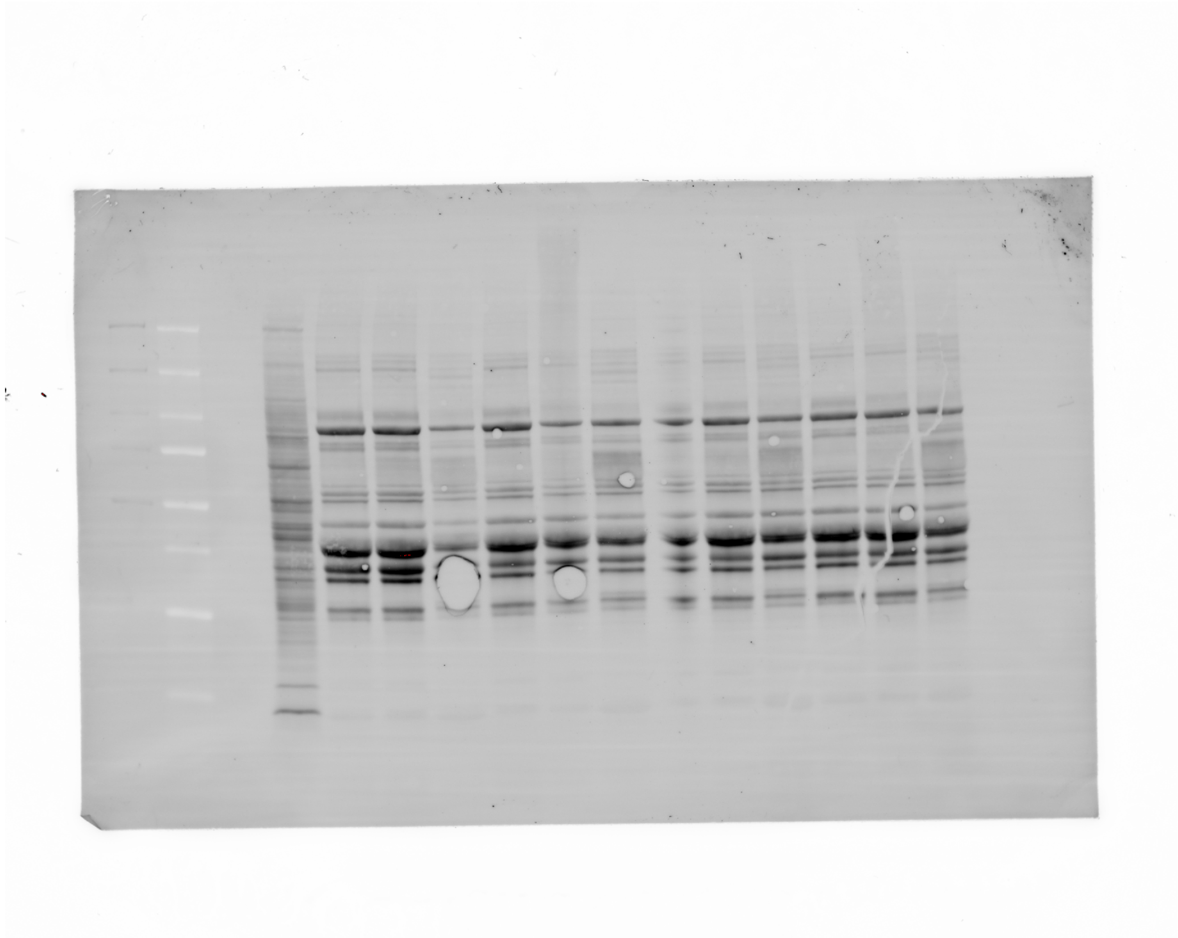

Adiponectin
Chemiluminescence blot 1
Figure 6E

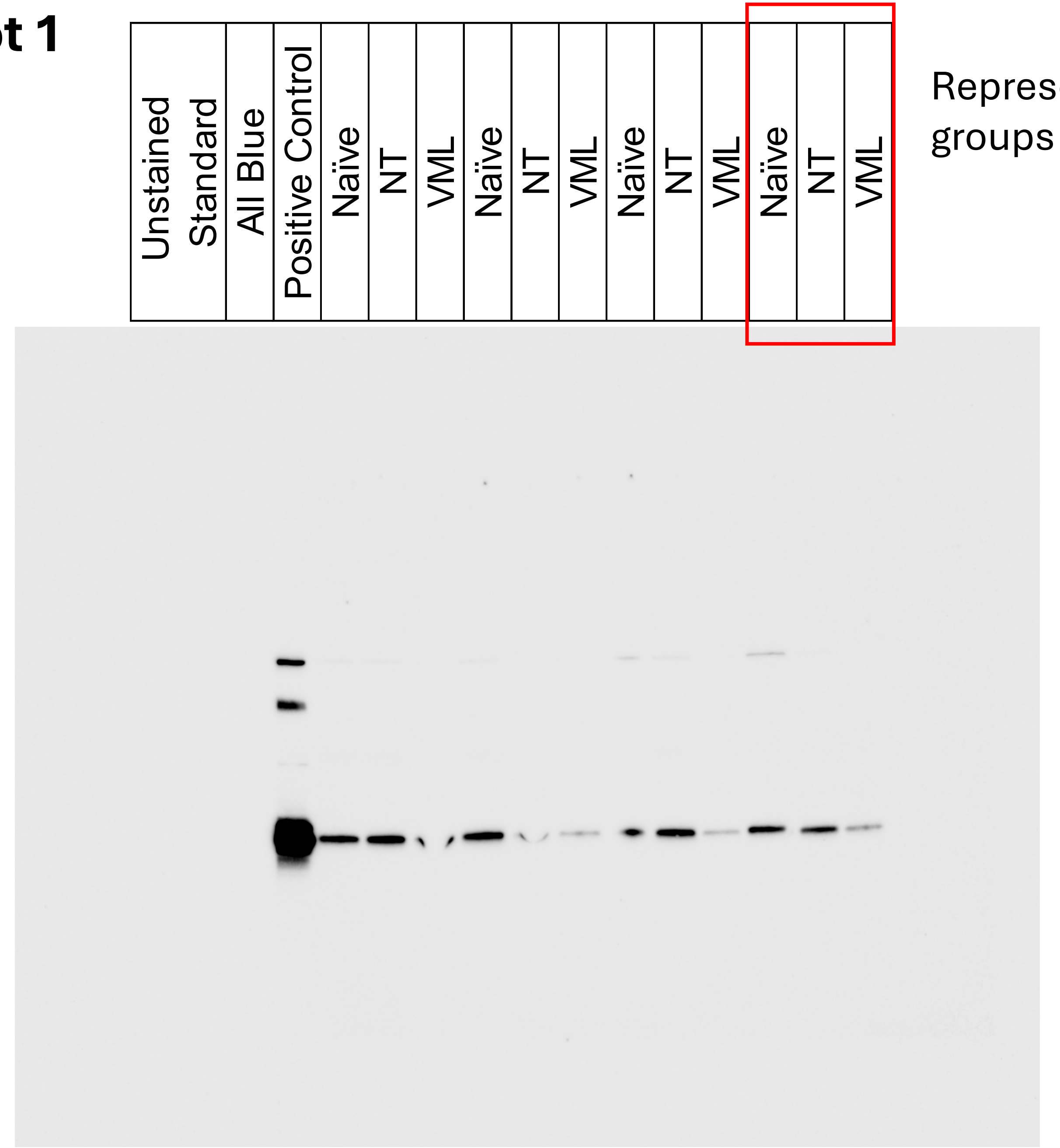

Representative images and groups are marked with red box

Adiponectin
Stain free gel 1
Figure 6E

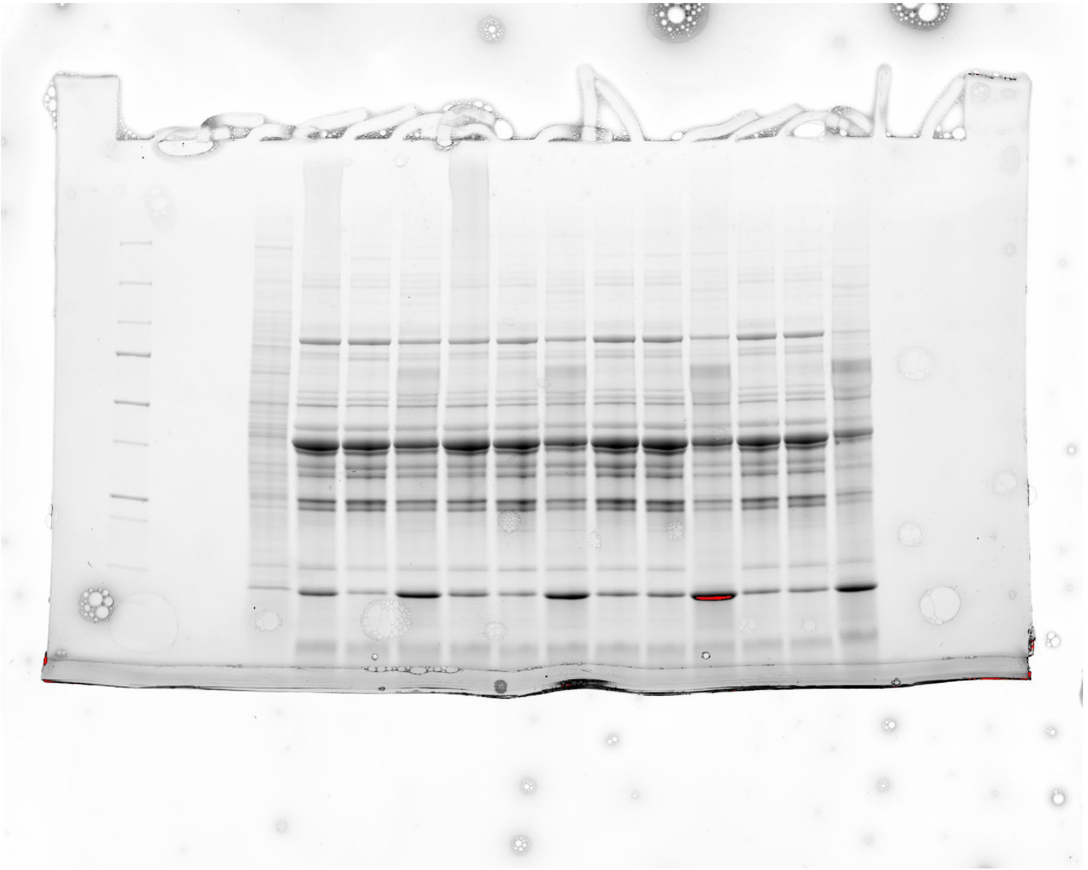

Adiponectin
Stain free blot 2
Figure 6E

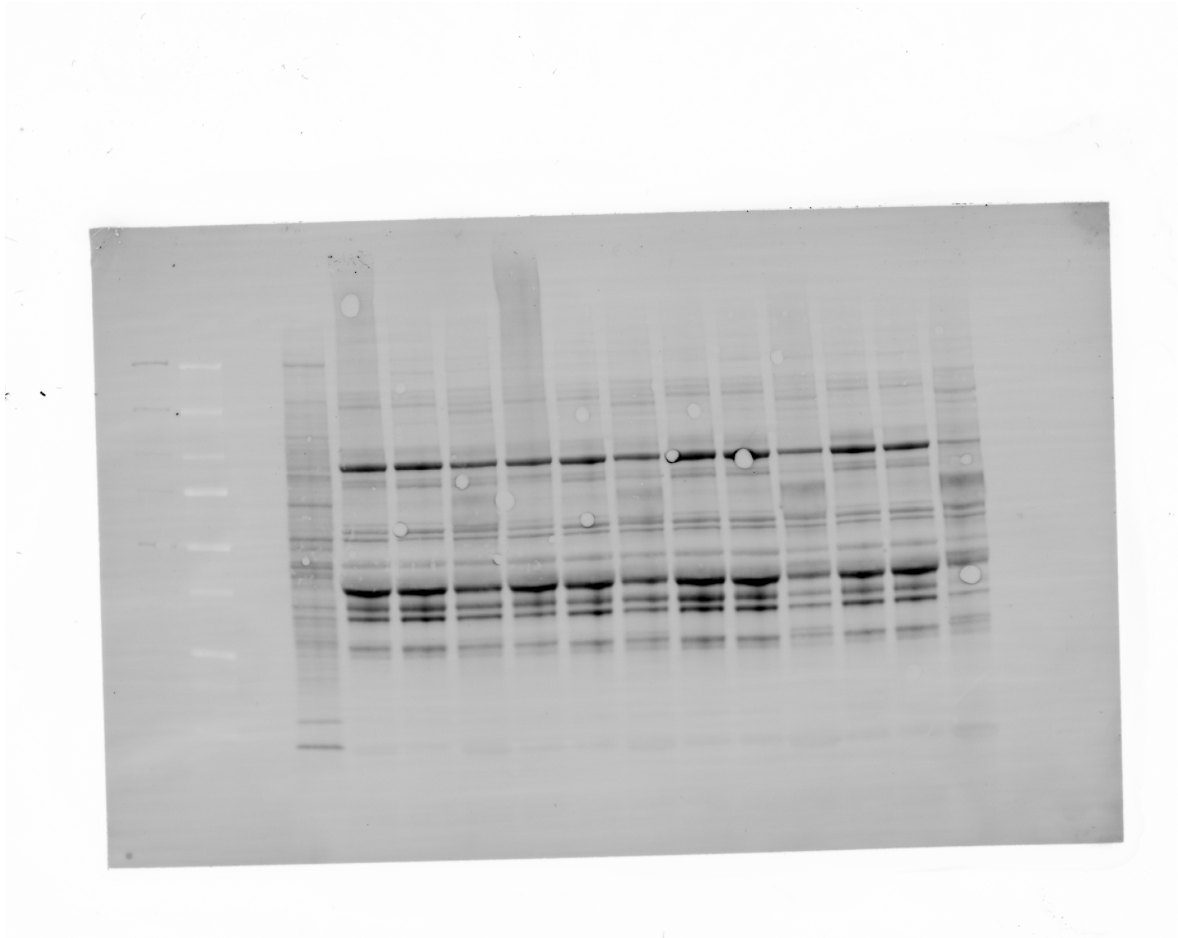

Adiponectin
Chemiluminescence blot 2
Figure 6E

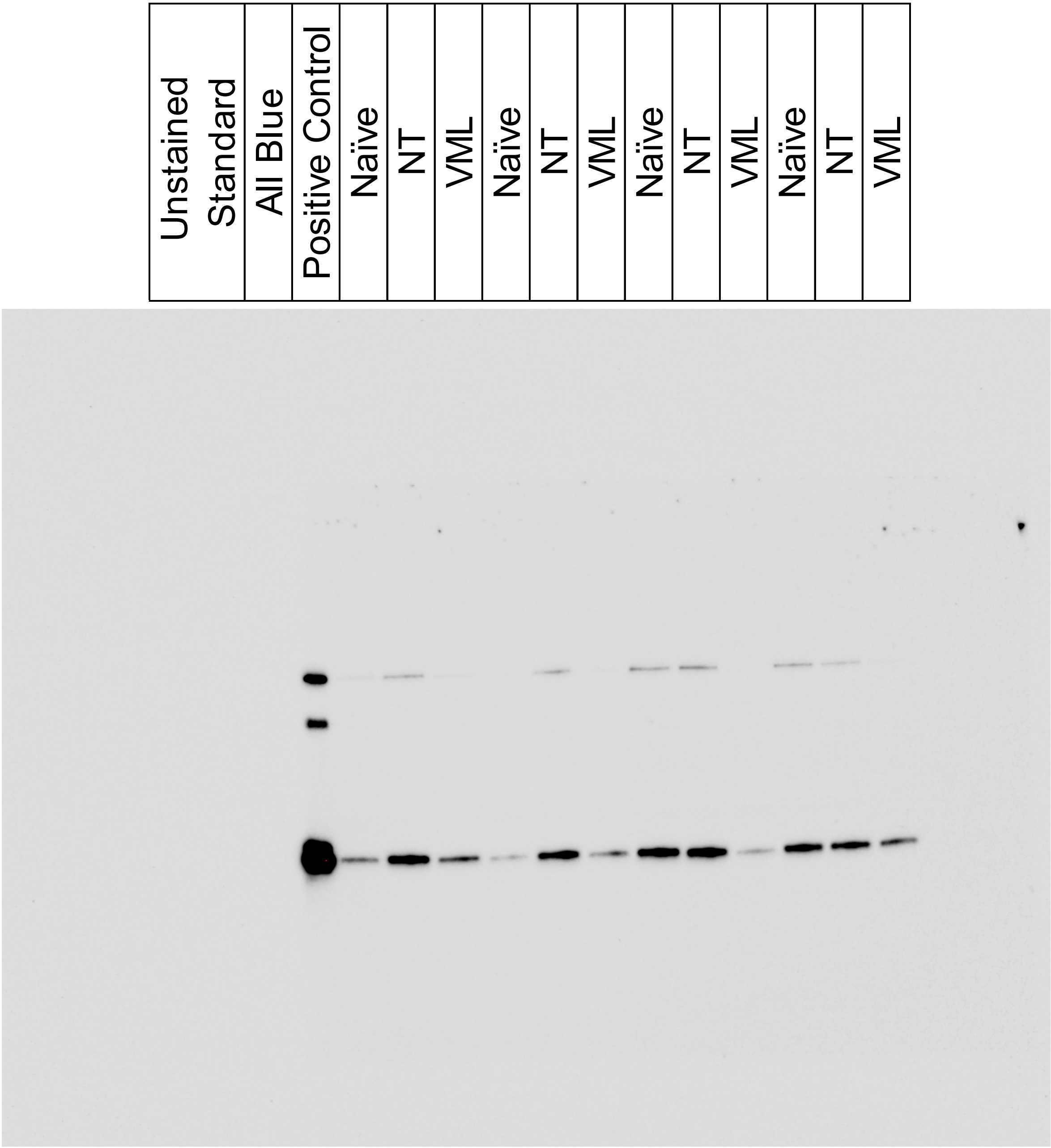

Perilipin 2
Stain free gel 1
Figure 6F

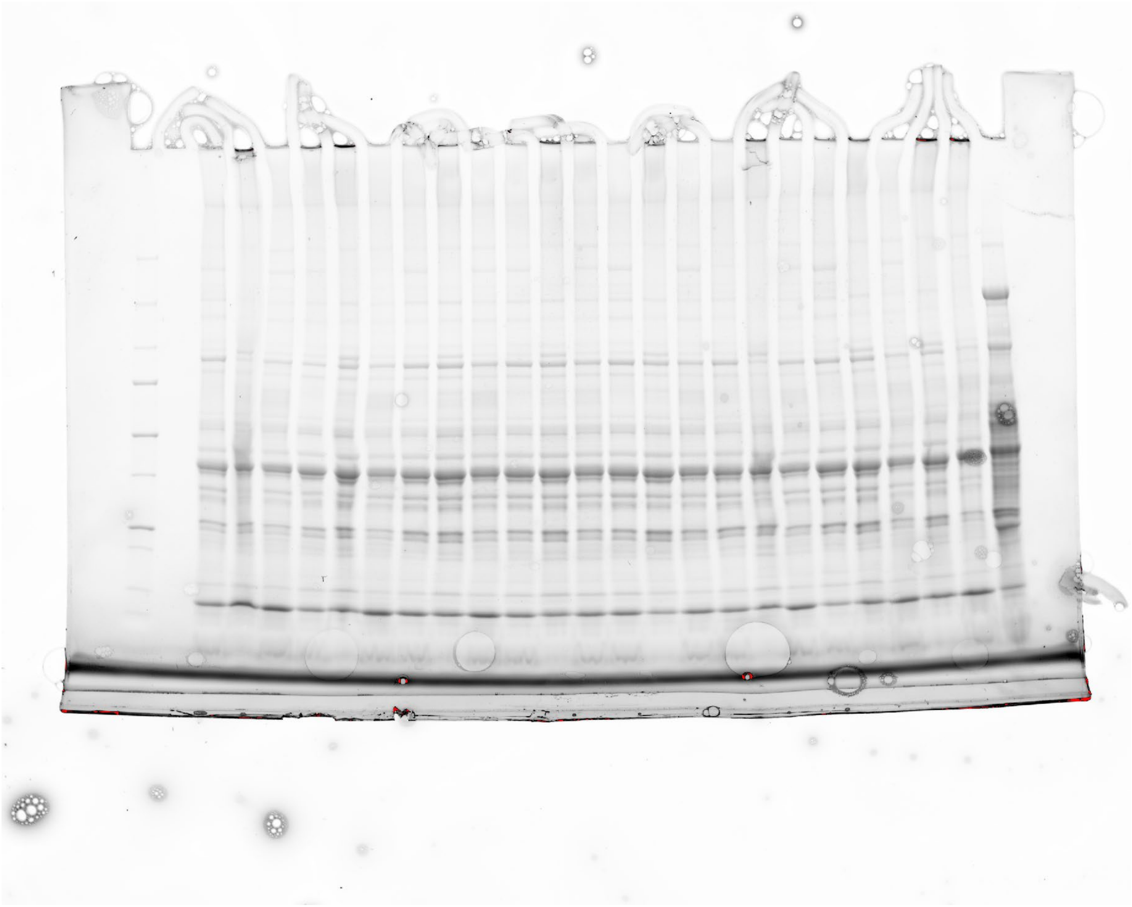

Perilipin 2
Stain free blot 1
Figure 6F

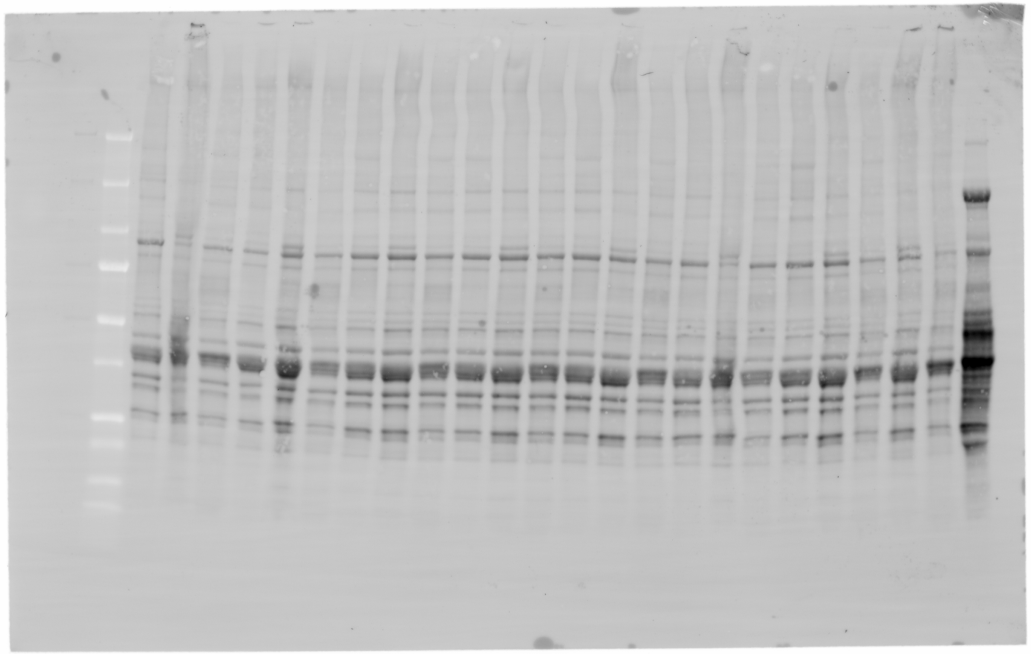

Perilipin 2
Chemiluminescence blot 1
Figure 6F

Representative images and groups are marked with red box

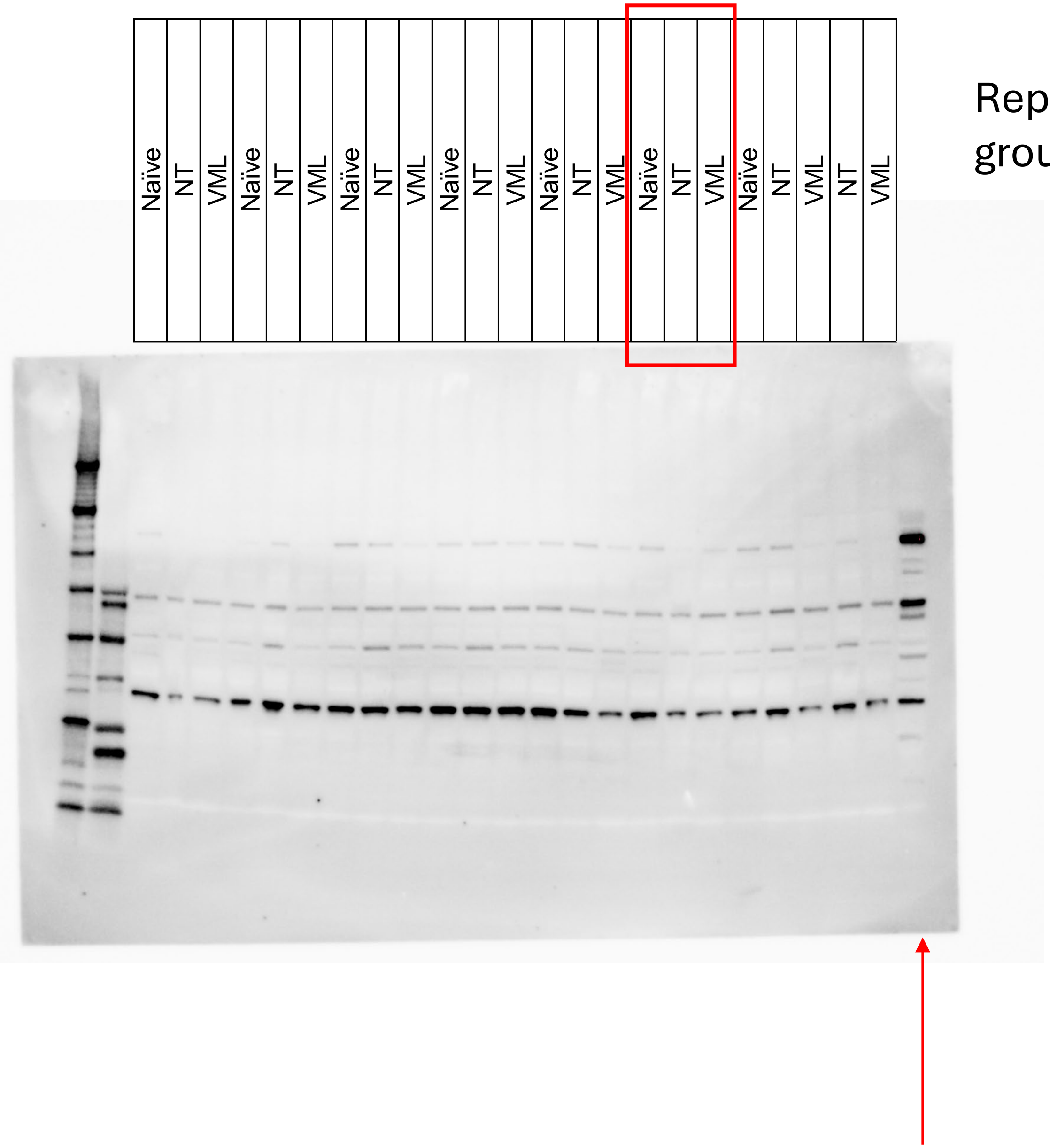

Practice sample
and not included
in analysis

Perilipin 5
Stain free gel 1
Figure 6G

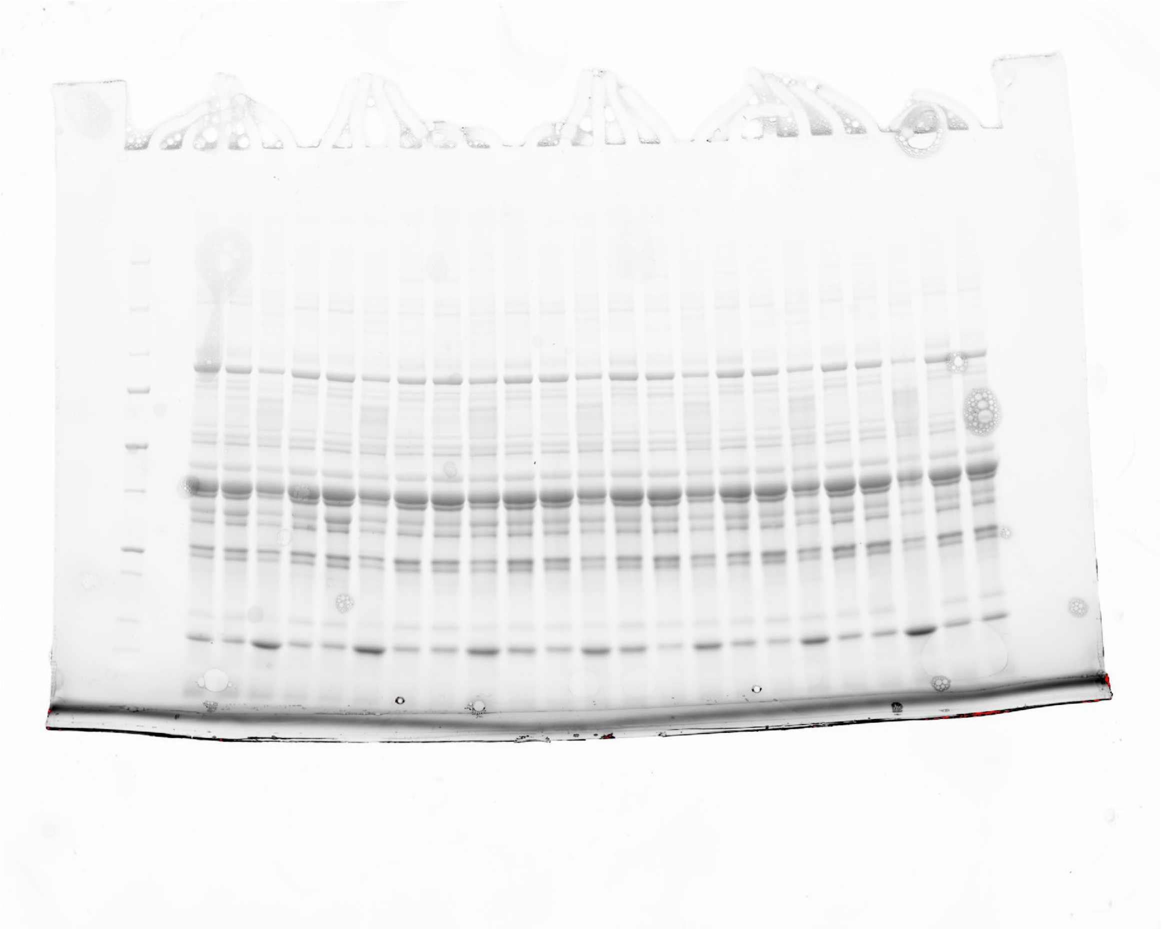

Perilipin 5
Stain free blot 1
Figure 6G

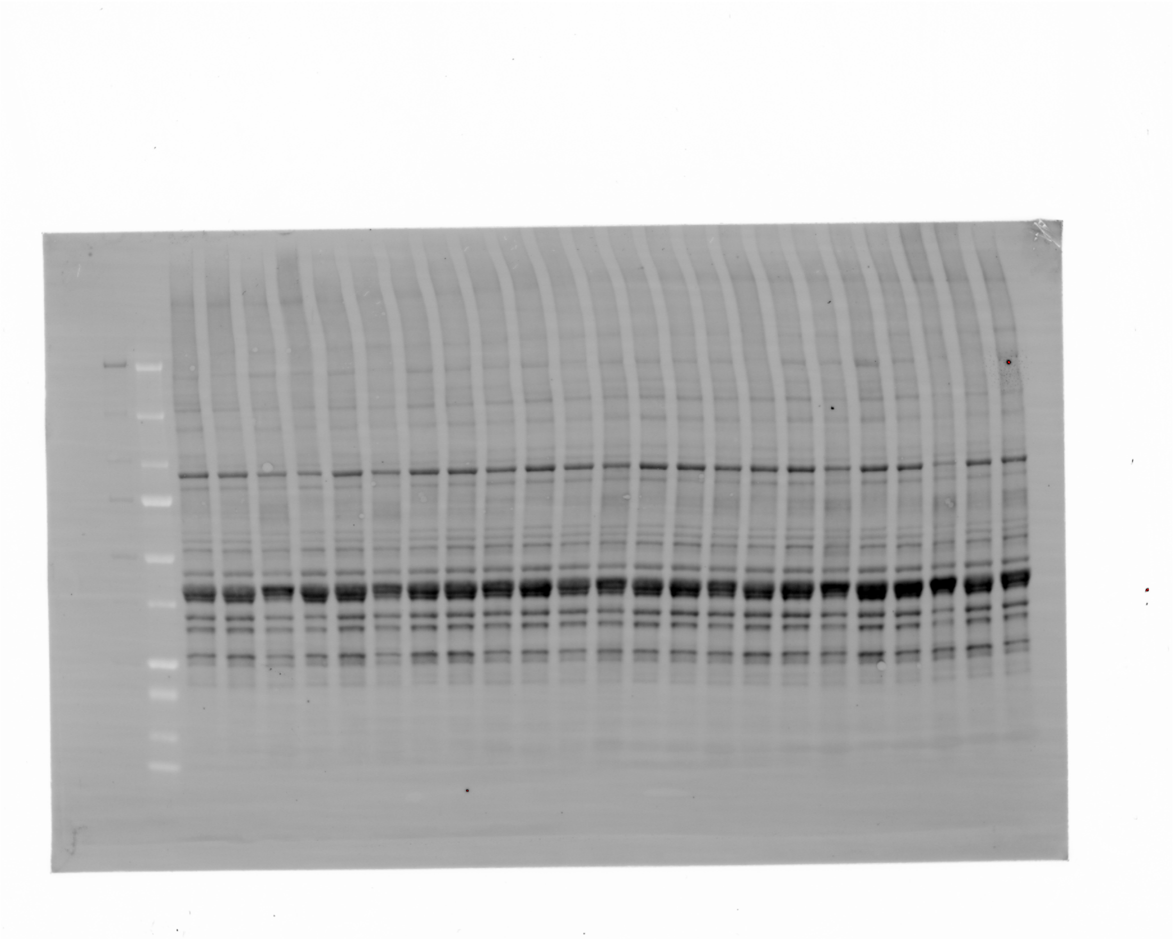

Perilipin 5
Chemiluminescence blot
Figure 6G

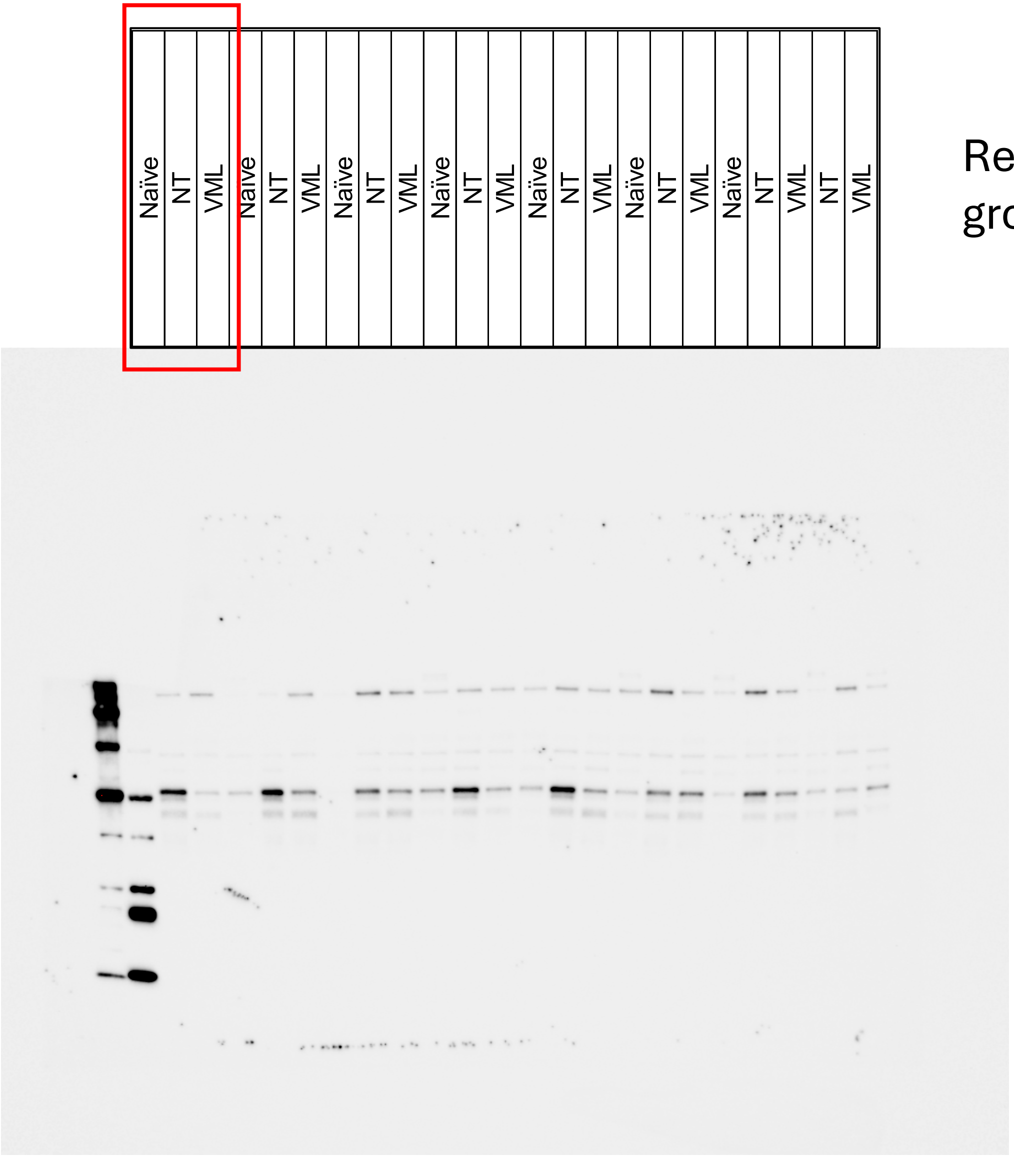

Representative images and groups are marked with red box
